# Integrative Multi‐Omics Approaches Reveal Selectivity Profiles and Molecular Mechanisms of FIIN‐2, a Covalent FGFR Inhibitor

**DOI:** 10.1002/advs.202412578

**Published:** 2025-02-20

**Authors:** Ying Fu, Dandan Zhu, Xiaojuan Chen, Lingzhi Qu, Ming Guo, Shuhong Zhang, Guangyu Xu, Zhuchu Chen, Maoyu Li, Yongheng Chen

**Affiliations:** ^1^ Department of Oncology NHC Key Laboratory of Cancer Proteomics and State Local Joint Engineering Laboratory for Anticancer Drugs National Clinical Research Center for Geriatric Disorders Xiangya Hospital Central South University Changsha Hunan 410008 China; ^2^ Key Laboratory of Chemical Biology and Traditional Chinese Medicine Ministry of Educational of China Key Laboratory of the Assembly and Application of Organic Functional Molecules of Hunan Province College of Chemistry and Chemical Engineering Hunan Normal University Changsha Hunan 410081 China

**Keywords:** AMPKα1, chemoproteomics, FGFR inhibitor, HCC, multi‐omics

## Abstract

Fibroblast growth factor receptor (FGFR) inhibitors are emerged as an important class of targeted therapies in oncology, targeting key pathways associated with tumor growth, angiogenesis, and resistance to conventional treatments. FIIN‐2, the first irreversible covalent pan‐FGFR inhibitor, has shown promise in overcoming resistance due to gatekeeper mutations; however, its selectivity and molecular mechanisms in tumors remain poorly understood. In this study, an FIIN‐2 chemical probe is designed and synthesized to identify both established and novel targets in hepatocellular carcinoma (HCC) via chemoproteomic profiling. An integrative multi‐omics approach, including chemoproteomic, phosphoproteomic, transcriptomic, and proteomic analyses, is utilized to elucidate the full spectrum of target proteins, signaling pathways, and downstream effectors regulated by FIIN‐2 in HCC. Notably, adenosine monophosphate‐activated protein kinase α1 (AMPKα1) is identified as a novel target of FIIN‐2, with Cys185 identified as its covalent binding site. These findings reveal that FIIN‐2 can induce autophagy by directly binding to and activating AMPKα1, influencing its anti‐tumor activity in HCC cells. Overall, this study greatly advances the understanding of FIIN‐2′s on‐ and off‐target effects, offering a comprehensive view of its molecular mechanisms in cancer cells. The integrative multi‐omics approach provides a valuable framework for the development and optimization of covalent kinase inhibitors.

## Graphical abstract

Using integrative multi‐omics analyses, this study maps FIIN‐2's targets and signaling networks in liver cancer cells, and identifies covalent AMPKα1 binding as a novel autophagy‐activating mechanism. The findings resolve both on‐target FGFR inhibition and off‐target protein engagement, providing a molecular roadmap to refine covalent kinase inhibitors for improved specificity and therapeutic outcomes.

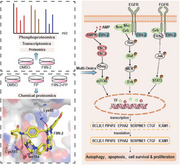

## 1 Introduction

Fibroblast growth factor receptors (FGFRs), one of the most important subfamilies of receptor tyrosine kinases (RTKs), play critical roles in various physiological and pathological processes, such as embryonic development, cell proliferation, differentiation, migration, apoptosis, and angiogenesis.^[^ ^1^ ^]^ Human FGFRs consist of four highly conserved members (FGFR1‐4), which are activated through binding with 22 different FGF ligands, subsequently inducing dimerization and intracellular autophosphorylation and triggering the activation of downstream signaling pathways, including the phosphoinositide 3‐kinase/protein kinase B (PI3K/AKT) and rat sarcoma/rapidly accelerated fibrosarcoma/mitogen‐activated protein kinase (RAS/RAF/MAPK) pathways.^[^ ^2^ ^]^ FGFR aberrations are common in multiple tumor types, and dysregulation of the FGF/FGFR signaling pathway has been closely implicated in the tumorigenesis, progression, and metastasis of various tumors, especially hepatocellular carcinoma (HCC).^[^ ^3^ ^]^ Increasing evidence has demonstrated that FGF19 amplification or overexpression and its counterpart receptor FGFR4 overexpression occur in 20–30% of HCC patients,^[^ ^4^ ^]^ and aberrant FGF19/FGFR4 signaling activation is considered an oncogenic driver of HCC.^[^ ^5^ ^]^ Thus, targeting FGFRs has emerged as a promising therapeutic strategy for tumors, including HCC.^[^ ^6^ ^]^

In recent years, an increasing number of small molecular FGFR inhibitors have been developed, and multiple pan‐FGFR inhibitors have been in clinical trials for the treatment of FGFR‐driven tumors; among them, Erdafitinib and Pemigatinib have already been approved for treating advanced or metastatic urothelial carcinoma,^[^ ^7^ ^]^ and metastatic cholangiocarcinoma,^[^ ^8^ ^]^ respectively. Several selective FGFR4 inhibitors, such as BLU554,^[^ ^9^ ^]^ H3B6527,^[^ ^10^ ^]^ and FGF401,^[^ ^11^ ^]^ are under HCC clinical investigation. Notably, the above three selective FGFR4 inhibitors are covalent inhibitors. Covalent kinase inhibitors have attracted intensive attention for their better binding affinity and prolonged pharmacodynamic effects. Specifically, there is a reactive functional group, otherwise known as the warhead, in covalent kinase inhibitors, which can form a covalent interaction with a certain residue of the target kinase, thus improving the binding affinity and duration. Moreover, compared with noncovalent inhibitors, covalent kinase inhibitors display better performance against drug resistance caused by mutations.^[^ ^12^ ^]^ Nonetheless, inadvertent or excessive binding with undesired target proteins could lead to uncontrolled cell toxicity, which is the greatest obstacle hindering the development of covalent kinase inhibitors. Similarly, unexpected off‐target effects and side effects hamper the clinical application of FGFR covalent inhibitors.^[^ ^13^ ^]^ Therefore, an in‐depth study of the selectivity, proteome‐wide binding targets, and potential off‐target effects of FGFR covalent inhibitors would deepen our understanding of the mechanism of FGFR inhibitors, facilitate their further improvement, and enhance the clinical benefits.

Over the past decade, a chemical proteomic strategy represented by activity‐based protein profiling (ABPP) has emerged as one of the most effective methods for identifying the direct target proteins of bioactive small molecules, especially covalent kinase inhibitors.^[^ ^14^ ^]^ Functional chemical probes are usually obtained by appending a terminal alkyne group to covalent inhibitors. Through the copper‐catalyzed azide‐alkyne cycloaddition (CuAAC) reaction, also known as “click chemistry,” alkynes can achieve in‐gel visualization or pull‐down of tagged proteins via conjugation with rhodamine‐azide or biotin‐azide, respectively. Furthermore, combined with quantitative mass spectrometry (MS), it can identify the global proteomic profile of covalent inhibitors in living systems. In addition, considering that kinase inhibitors act mainly by regulating the phosphorylation activity of target kinases as well as downstream signaling, phosphoproteomic studies enable us to elucidate the global phosphorylation changes caused by inhibitors. Proteomics and transcriptomics, on the other hand, could reveal the final effectors induced by the inhibitors. Therefore, the integration of chemical proteomics with multi‐omics approaches, such as phosphoproteomics, proteomics, and transcriptomics analysis, would yield a comprehensive elucidation of the diverse targets and mechanisms underlying the effects of covalent kinase inhibitors.

FIIN‐2 was the first, next‐generation, irreversible‐covalent FGFR inhibitor that displayed great affinity for FGFR subtypes, including FGFR4. The crystal structure of the FGFR4 kinase domain with FIIN‐2 revealed that the reactive acrylamide group of FIIN‐2 can form a covalent bond with Cys477 in the kinase P‐loop and displays a “DFG‐out” conformation, which enables it to avoid steric clashes with the gatekeeper mutation.^[^ ^15^ ^]^ In addition to FGFRs, FIIN‐2 has been demonstrated to be moderately potent against the epidermal growth receptor (EGFR), proto‐oncogene tyrosine‐protein kinase Src (SRC), and tyrosine‐protein kinase Yes (YES).^[^ ^16^ ^]^ However, it is unclear whether there are other on‐target engagements and off‐target activities of FIIN‐2. Thus, identification of the proteome‐wide binding profile and off‐target proteins of FIIN‐2 is imperative for studies of covalent FGFR inhibitors in HCC.

In this study, we developed a functional chemical probe of FIIN‐2 and assessed its biological activity in HCC. Using an MS‐based chemical proteomics approach, we identified numerous potential target proteins of FIIN‐2 in HCC cells and subsequently confirmed that adenosine monophosphate‐activated protein kinase α1 (AMPKα1) is a novel target protein of FIIN‐2, and Cys185 was validated as the covalent binding site. Moreover, employing multi‐dimensional integration analysis and validation encompassing chemical proteomics, phosphoproteomics, proteomics, and transcriptomics datasets enabled us to construct a comprehensive action network of FIIN‐2 in HCC. These findings offer valuable insights for the further advancement and optimization of FGFR inhibitors in targeted therapy against tumors.

## 2 Results

### 2.1 Design and Evaluation of the FIIN‐2 Probe (FP)

**Figure 1:**
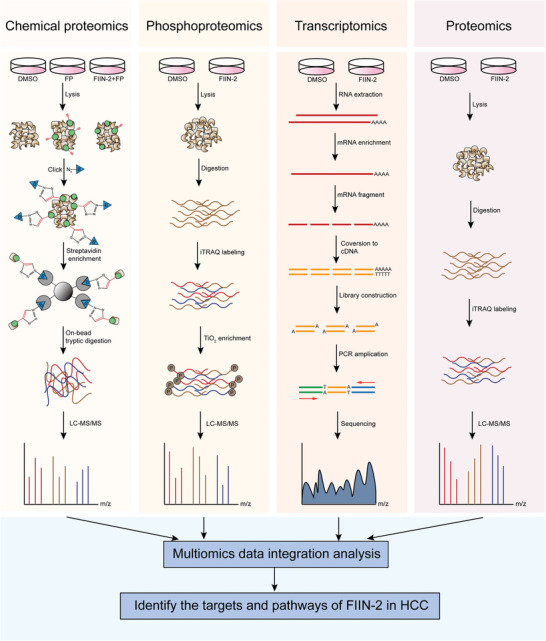
Schematic of the multi‐omics strategy used to identify the targets and pathways of FIIN‐2 in HCC. The detailed procedures of chemical proteomics, phosphoproteomics, transcriptomics, and proteomics are shown in the figure.

**Figure 2:**
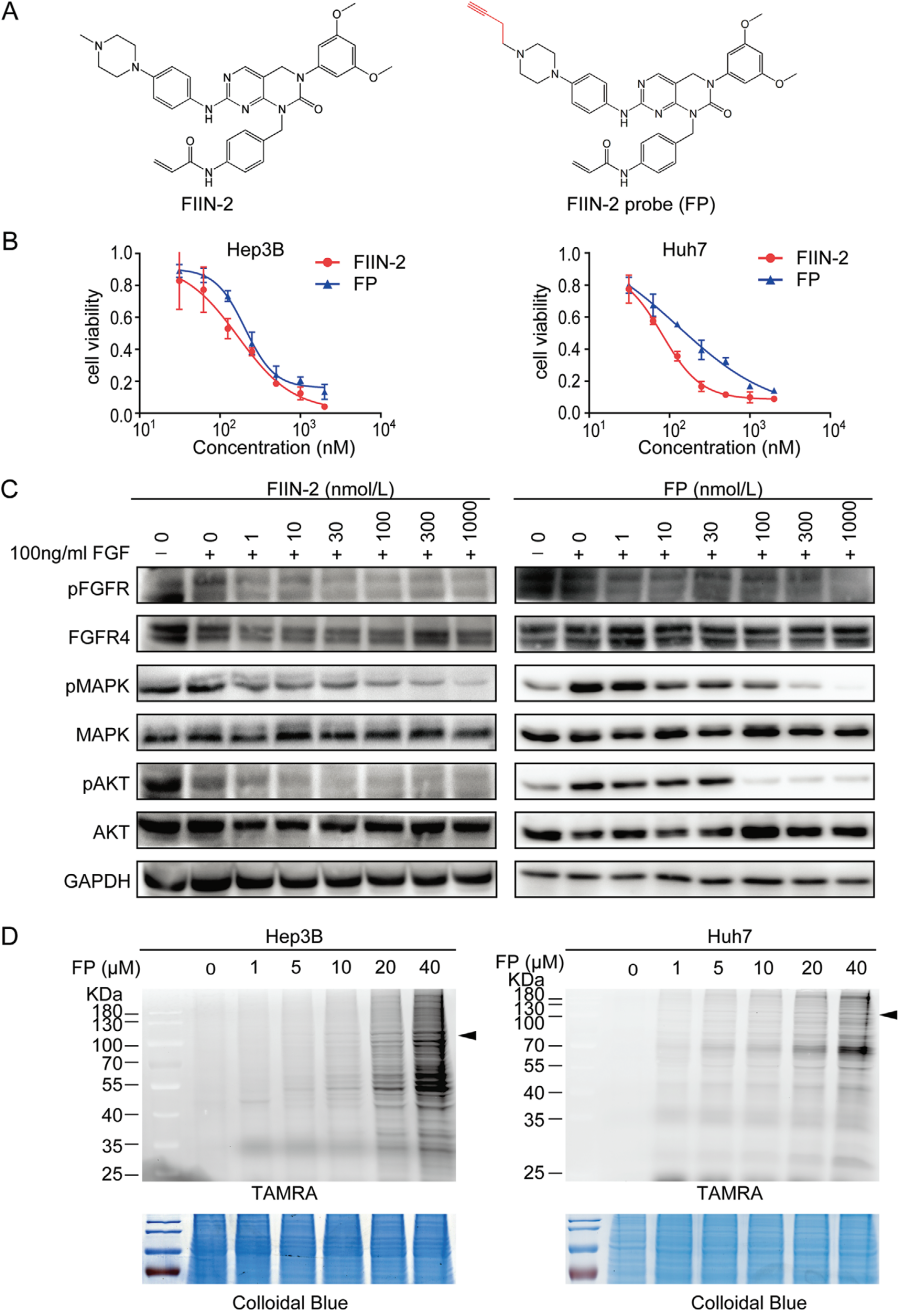
Biological evaluation of the activity of FP. A) Chemical structures of FIIN‐2 and FP. B) The viability of Hep3B and Huh7 cells treated with FIIN‐2 and FP was measured *via a* CCK‐8 assay. C) The FGFR downstream signaling pathway was inhibited by FIIN‐2 and FP in a similar concentration‐dependent manner. D) Gel‐based ABPP reveals the proteome reactivity of Hep3B and Huh7 cells labeled *with* FP. Coomassie blue staining was used as a loading control.

To explore the complete target proteins, signaling pathways, and relevant changes in the whole transcriptome and proteome caused by FIIN‐2 in HCC, we employed a multi‐omics study including chemical proteomics, phosphoproteomics, transcriptomics, and proteomics in HCC cells treated with FIIN‐2 and conducted multi‐dimensional data integration analysis (**Figure** **1**). To identify the target protein profile of FIIN‐2 using ABPP‐based chemical proteomics, an alkyne‐tagged probe for FIIN‐2 was first designed and synthesized (**Figure** **2**A). Cell viability assays were performed to verify the inhibitory effect of FP, and the results showed that similar to FIIN‐2, FP suppressed cell proliferation in Hep3B and Huh7 cells (Figure 2B). Cell proliferation and colony formation experiments were also performed to further validate the similar inhibitory effects of FIIN‐2 and FP (Figure S1, Supporting Information). Furthermore, FIIN‐2 and FP inhibited FGFR and its downstream signaling pathway, including the phosphorylation of MAPK and AKT, in a concentration‐dependent manner (Figure 2C). These results indicated that the addition of an alkyne group to FIIN‐2 had no significant effect on its activity and that FP almost preserves the same biological function as FIIN‐2. Moreover, as it possesses an alkyne tag, FP enables the labeling of its covalently bound target proteins and their visualization by reacting with rhodamine‐azide through the CuAAC reaction. As shown in Figure 2D, the FGFRs could be distinctly visualized by FP, and the labeled proteins obviously increased with increasing concentrations of FP.

### 2.2 Selectivity Proteomic Profiling of FIIN‐2 by Chemical Proteomics

**Figure 3:**
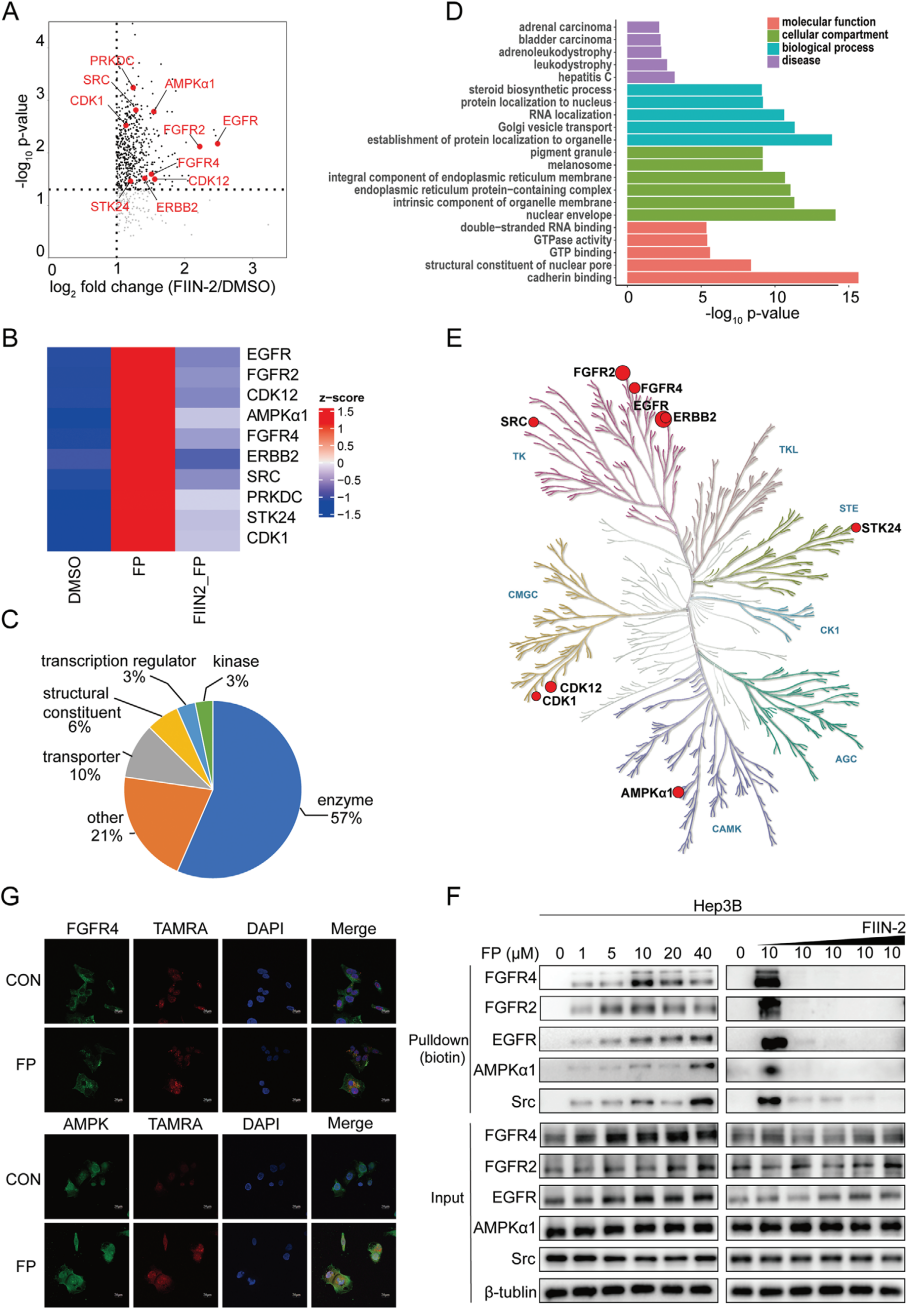
Activity‐based protein profiling identified potential protein targets of FIIN‐2. A) Volcano plot for the quantification of ABPP. Only proteins showing a fold change in enrichment greater than 2 in competition experiments (FIIN‐2‐probe/DMSO) were considered. The black dotted proteins represent the 422 candidate target proteins. The red dots represent the top 10 candidate target kinases. B) The top 10 FIIN‐2‐targeted kinases. C) Functional classification of FIIN‐2‐targeted proteins. D) Gene ontology and disease analysis of FIIN‐2‐targeted proteins. E) Classification of FIIN‐2‐targeted kinases. F) Pull‐down validation of FIIN‐2‐targeted proteins. G) Confocal fluorescence imaging of Hep3B cells treated with FP and anti‐FGFR4 or AMPKα1 antibodies. Blue: DAPI nuclear staining. Green: FGFR4 or AMPKα1 staining. Red: TAMRA channel. Scale bar: 20 µm.

To assess the on‐target engagement and off‐target activity of FIIN‐2, a chemical proteomics strategy based on MS was conducted on Hep3B cells treated with DMSO or FP. For competitive ABPP experiments, cells were treated with FP after pretreatment with FIIN‐2. The proteins labeled by FP were subsequently conjugated with biotin azide through a click chemistry reaction, enriched by streptavidin beads, and identified by liquid chromatography‐tandem mass spectrometry (LC‐MS/MS). A total of 2742 proteins were identified in all three groups (Table S1, Supporting Information). The target proteins were then filtered according to the following criteria: 1) fold change >2 (FP compared to DMSO, FP compared to FIIN2+FP); 2) statistical *p* value less than 0.05. Finally, 422 proteins were identified as potential targets of FIIN‐2 in Hep3B cells (Table S2, Supporting Information). As expected, FGFR2 and FGFR4 were identified among the top 10 kinases (**Figure** **3**A,B). FGFR1 and FGFR3 were not included in the list, possibly due to their low expression in Hep3B cells. Among the other eight identified kinases, EGFR and SRC have been verified to covalently bind to FIIN‐2,^[^ ^15^, ^16^ ^]^ but others have not been reported.

An analysis of protein annotations revealed that these FIIN‐2‐targeting proteins included enzymes, transporters, transcription regulators, kinases, and so on (Figure 3C). Further gene ontology (GO) and kyoto encyclopedia of genes and genomes (KEGG) analyses indicated that the identified target proteins were involved mainly in cadherin binding, GTP binding, and GTPase activity. Notably, these proteins are related to several types of human cancer (Figure 3D). In addition to kinases, the potential target proteins of FIIN‐2 included some nonkinase proteins that were enriched mainly in metabolism‐related pathways, such as fatty acid metabolism (Figure S2, Supporting Information). Furthermore, constructing the kinase profile for the identified kinases revealed that FGFRs, EGFR, receptor tyrosine‐protein kinase erbB‐2 (ERBB2), and SRC belong to the same kinase clade whose binding sites have been reported; however, the significantly identified AMPKα1 was distinctly different from them and caught our attention (Figure 3E). To further verify the true binding of these kinases identified via chemical proteomics, an in situ pulldown assay was performed through FP‐conjugated proteins reacting with biotin‐azide, and immunoblotting was performed with the corresponding antibodies. The amount of conjugated target proteins increased with increasing concentrations of FP, and the binding of these target proteins with FP could be competitively blocked by pretreatment with FIIN‐2 in Hep3B (Figure 3F) and Huh7 cells (Figure S3, Supporting Information). Moreover, the in situ labeling of FGFR4 and AMPKα1 by FP in Hep3B cells was demonstrated via an immunofluorescence assay combined with click chemistry reactions (Figure 3G).

### 2.3 Phosphoproteomic Analysis Reveals that Phosphorylation Profiling is Affected by FIIN‐2 in HCC

To gain insight into the phosphorylation profile and underlying mechanisms of FIIN‐2, we performed isobaric tags for relative absolute quantitation (iTRAQ)‐labeled quantitative phosphoproteomic analyses in Hep3B cells upon FIIN‐2 treatment. After normalization and redundancy removal, phosphoproteomic analysis revealed 10574 phosphosites, among which 84.1% were phospho‐Ser. A total of 894 phosphosites (fold change >1.2 and *p* value <0.05) were upregulated, and 895 phosphosites (fold change <0.83 and *p* value <0.05) were downregulated upon FIIN‐2 treatment (Table S3, Supporting Information).

**Figure 4:**
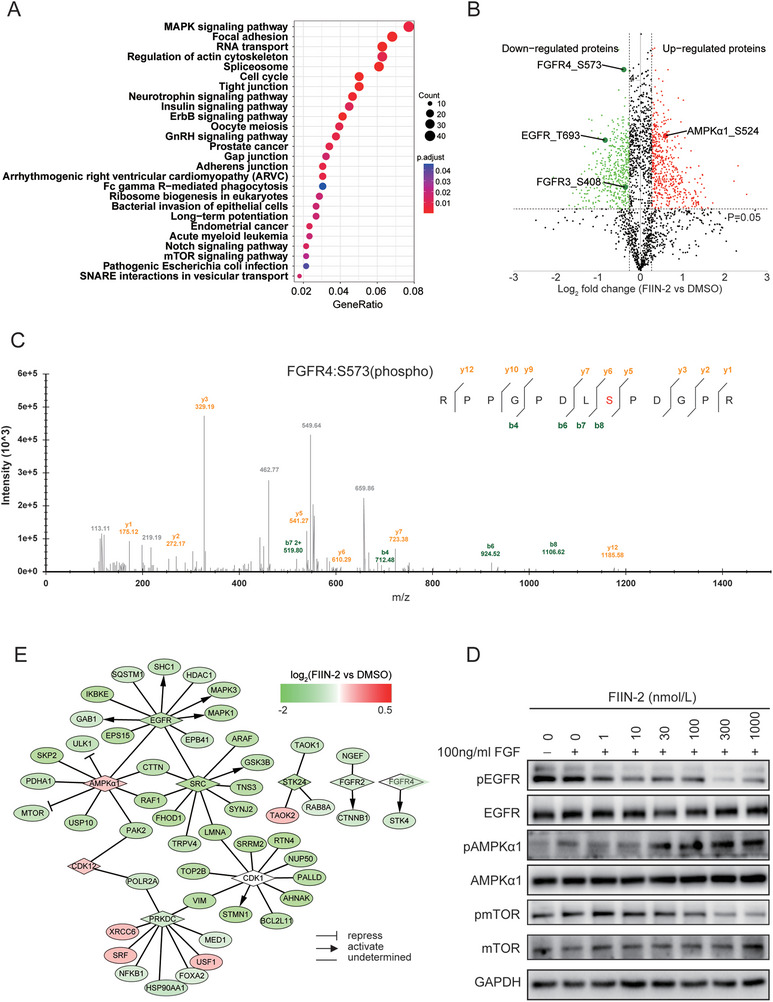
Quantitative phosphoproteomic analysis of Hep3B cells treated with FIIN‐2. A) KEGG pathway enrichment analysis of the differentially phosphorylated proteins. B) Volcano plot for quantification of differential phosphosites. Red dots represent upregulated genes, and green dots represent downregulated genes. C) Mass spectra for FGFR4 phosphorylation site identification. D) Validation of the identified phosphorylated proteins by western blotting. E) Network of kinases and their regulatory substrate proteins in the phosphoproteomic dataset.

KEGG enrichment analysis revealed that the dysregulated phosphorylated proteins were significantly enriched in multiple signaling pathways, including the MAPK, ErbB, and serine/threonine‐protein kinase (mTOR) signaling pathways (**Figure** **4**A). As expected, the phosphorylation levels of several representative proteins of the FGFR signaling pathway, including FGFR3, FGFR4, GRB2‐associated‐binding protein 2 (GAB2), son of sevenless (SOS), RAF proto‐oncogene serine/threonine‐protein kinase (RAF1), and AKT, were decreased after FIIN‐2 treatment. For example, the phosphorylation of FGFR4 at S573 and FGFR3 at S408 was decreased (Figure 4B), which was further confirmed by MS/MS (Figure 4C). In addition, the phosphorylation of other proteins in important signaling pathways during oncogenesis also changed. For example, multiple phosphorylation sites of EGFR were found to be downregulated upon FIIN‐2 treatment. The phosphorylation of EGFR at T693 was first found to be regulated by FIIN‐2 and even decreased by 50% (Figure 4B). Interestingly, we first found that phosphorylation of AMPK at S524 was upregulated upon FIIN‐2 treatment (Figure 4B). The altered phosphorylation of AMPK and its canonical downstream protein mTOR was verified by western blotting in Hep3B (Figure 4D) and Huh7 cells (Figure S4, Supporting Information).

We further retrieved the substrates of the kinases in our phosphorylation dataset by querying PhosphoSitePlus (www.phosphosite.org) and constructed a network of kinases and their substrates via Cytoscape (Figure 4E). The phosphorylation of many substrates was altered in our phosphorylation dataset, possibly because FIIN‐2 regulated upstream kinase activity. For example, the phosphorylation of substrates of EGFR and FGFR was inhibited by FIIN‐2. mTOR and serine/threonine‐protein kinase ULK1 (ULK1), whose phosphorylation has been reported to be repressed by AMPK, were also downregulated upon FIIN‐2 treatment. Overall, these results are in line with the known inhibitory effect of FIIN‐2, indicating that FIIN‐2 influences many aspects of cell biological processes by targeting multiple kinases.

### 2.4 Transcriptomics and Proteomics Reveal the Downstream Regulatory Targets of FIIN‐2

**Figure 5:**
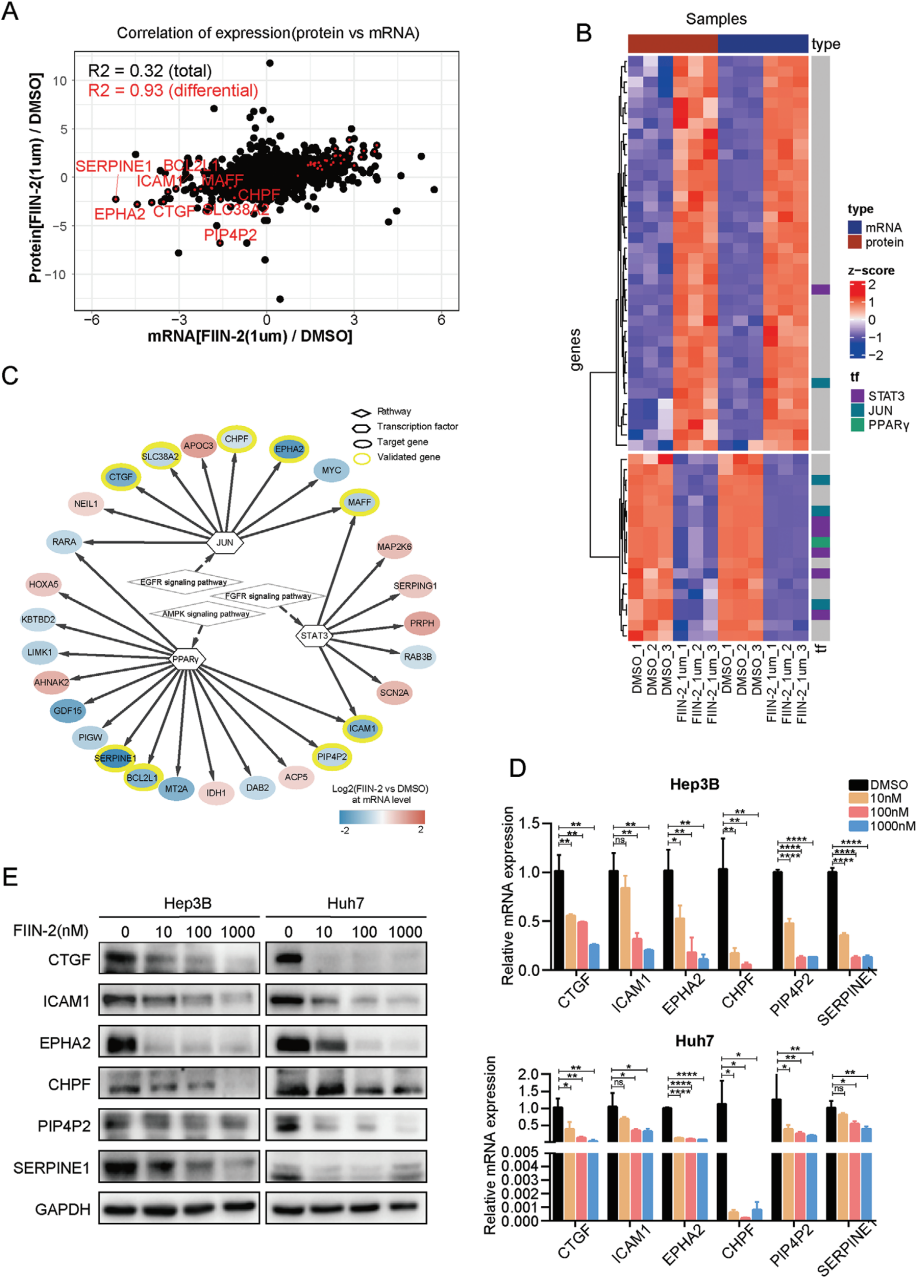
Transcriptome and proteome analyses revealed the downstream regulatory targets of FIIN‐2. A) Correlation analysis of the differentially expressed mRNAs and differentially expressed proteins induced by FIIN‐2. B) Heatmap of differentially expressed mRNAs and proteins. C) Regulatory network of the kinase pathway, transcription factors, and mRNAs or proteins. D) Validation of differential mRNA expression by qPCR. E) Validation of differential protein expression by Western blotting.

To explore the overall molecular changes resulting from FIIN‐2 treatment, we integrated transcriptomics and proteomics analyses. In the RNA‐seq study, 18 234 genes were quantified in all three biological replicates, and the expression of 2451 (13.4%) genes significantly changed upon FIIN‐2 treatment: 1454 genes (fold change > 1.5, *p* value < 0.05) were upregulated, and 997 genes (fold change < 0.67, *p* value < 0.05) were downregulated (Table S4, Supporting Information). In the proteomic study, 8104 proteins were quantified in all three biological replicates; a total of 216 proteins (fold change > 1.2, *p* value < 0.05) were upregulated, and 186 proteins (fold change < 0.83, *p* value < 0.05) were downregulated (Table S5, Supporting Information). Overall, only 6.8% (402) of the total protein content significantly changed upon FIIN‐2 treatment, demonstrating that the protein abundance changed less globally than did the transcriptome. To elucidate the effects of FIIN‐2 at the transcriptional and translational levels in an unbiased manner, we performed a correlation analysis of mRNA and protein expression patterns. A total of 7987 mRNA‒protein pairs were retrieved from our transcriptomic and proteomic datasets. By correlation analysis, we found that mRNA and protein expression were only moderately correlated at the overall level (Pearson correlation coefficient, *R* ^2^ = 0.32), whereas a strong correlation between mRNA and protein expression was found at the differential expression level (*R* ^2^ = 0.93) (**Figure** **5**A; Table S6, Supporting Information). By integrating transcriptomic and proteomic data, 91 genes were found to be differentially expressed at both the mRNA and protein levels, of which 86 genes changed consistently upon FIIN‐2 treatment (Figure 5B). As shown in Figure S5A (Supporting Information), there was a negative correlation between mRNA expression and protein expression after drug treatment. For objective analysis, we summarized these mRNAs and their corresponding proteins. The mRNA and protein expression of 42 genes significantly changed after drug treatment, but the opposite trend was detected. This phenomenon is reflected in the large amount of proteomic data. Our analysis revealed that these proteins are mainly proteases, transcription‐related molecules, and membrane transport molecules from a functional perspective (Figure S5A, Supporting Information). Gene ontology annotation revealed that catalytic activity involves mainly molecular functional regulation, transcriptional regulation, etc., whereas biological process annotation revealed that it involves mainly metabolic processes, biological regulation, and stimulus responses (Figure S5B, Supporting Information).

To further clarify the FIIN‐2‐regulated signaling pathways that ultimately lead to changes in gene transcription and protein expression, we constructed a network of signaling pathways affected by FIIN‐2 and its downstream transcription factors (mainly including signal transducer and activator of transcription 3 [STAT3], transcription factor jun [JUN], and peroxisome proliferator‐activated receptor gamma [PPARγ]), and ultimately regulatory target genes by searching the transcription factor database (Figure 5C). We further verified these regulatory relationships by qPCR and western blotting. These results indicated that FIIN‐2 can decrease the expression of the downstream genes regulated by FGFR (ex. connective tissue growth factor [CTGF], intercellular adhesion molecule 1 [ICAM1]), EGFR (ex. ephrin type‐A receptor 2 [EPHA2], chondroitin sulfate synthase 2 [CHPF]), and AMPKα1 (ex. type 2 phosphatidylinositol 4,5‐bisphosphate 4‐phosphatase [PIP4P2], endothelial plasminogen activator inhibitorSerpin E1 [SERPINE1]) at both the mRNA and protein levels in a concentration‐dependent manner (Figure 5D,E).

### 2.5 FIIN‐2 Can Induce Autophagy in HCC Cells by Directly Targeting AMPKα1 at Cys185

**Figure 6:**
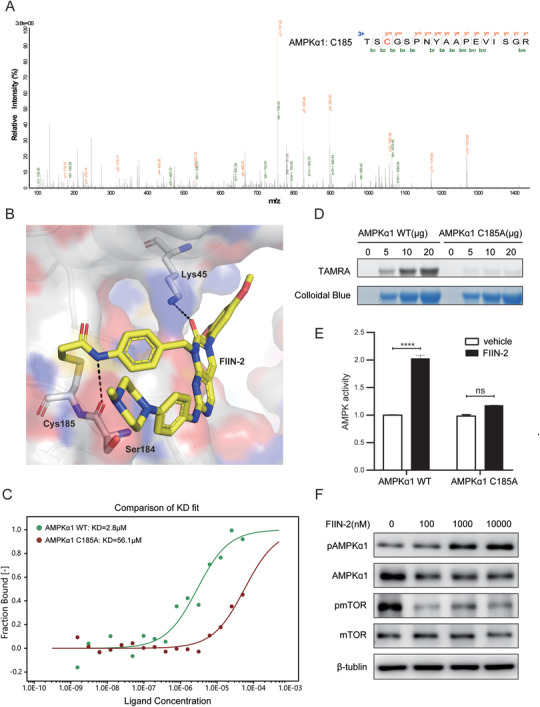
FIIN2 can target and activate AMPKα1 at Cys185. A) MS analysis revealed Cys185 of AMPKα1 as the binding site of FIIN‐2. B) Predicted binding model of FIIN‐2 and AMPKα1. The structure of AMPKα1 is shown as a surface with key residues labeled as gray sticks, and FIIN‐2 is displayed as a yellow stick. Hydrogen bonds are shown as black dashed lines. C) The binding affinities of AMPKα1 WT and AMPKα1 C185A for FIIN2 were analyzed via MST. D) In gel, ABPP shows FP can label recombinant wild‐type AMPKα1 but not mutant AMPKα1 C185A. E) Kinase assay showing the activation effect of AMPKα1 WT and AMPKα1 C185A by FIIN2. F) FIIN2 can activate AMPK signaling by elevating the phosphorylation of AMPK and inhibiting the phosphorylation of mTOR.

Chemical proteomic analysis and validation suggested that AMPKα1 is a novel target for FIIN2 in HCC. To identify the binding site between FIIN‐2 and AMPKα1, purified AMPK was incubated with FIIN‐2, and the complex was analyzed by MS. The results revealed that Cys185 in AMPKα1 was the binding site of FIIN‐2 (**Figure** **6**A). The modeled structure of AMPKα1/FIIN‐2 subsequently revealed that FIIN‐2 binds to the surface of the AMPKα1 activation loop, forming a covalent bond with Cys185 and hydrogen‐bonding interactions with Ser184 and Lys45 (Figure 6B). Furthermore, LC‐MS/MS was utilized to detect the covalent peptide‒drug adducts in vitro. The results revealed that the C185 site was identified after DMSO treatment (Figure S6A, Supporting Information). Under FIIN2‐treated conditions, the C185 site was identified as covalently bound to FIIN2 (Figure S6B, Supporting Information). Only the C185A site was identified after FIIN2 treatment, which appeared to disable the binding of FIIN2 to AMPKɑ1 at this site (Figure S6C, Supporting Information).

To validate the true binding between FIIN‐2 and Cys185 of AMPKα1, AMPKɑ1 (residues 1–312) and the mutant (C185A) were expressed and purified. The binding affinity between FIIN‐2 and AMPKα1 was evaluated via microscale thermophoresis (MST), and the results revealed that the binding affinity of FIIN‐2 with AMPKα1 WT was almost 20 times greater than that with AMPKα1 C185A (Figure 6C). The labeling efficiency of FP for AMPKα1 was assessed by in‐gel fluorescence. The results also revealed that FP could label AMPKα1 WT but not C185A and the labeled amounts increased with increasing protein content (Figure 6D). Given that AMPKα1 is the catalytic subunit of AMPK, we wondered whether FIIN2 affects its enzymatic activity. Therefore, we conducted a kinase assay, the results of which demonstrated that FIIN‐2 can significantly elevate the kinase activity of AMPKα1 WT but not C185A (Figure 6E). Consistently, western blotting revealed that FIIN‐2 enhanced the phosphorylation of AMPKɑ1 and decreased the phosphorylation of mTOR, which is downstream of the AMPK signaling pathway (Figure 6F), whereas the overexpression of AMPKα1 enhanced this effect (Figure S7A, Supporting Information). Additionally, the activation of AMPK signaling by FIIN2 has been demonstrated in multiple types of human cancer cells, such as pancreatic cancer (PANC), lung cancer (PC‐9), and breast cancer (MDA‐MB‐231) cells (Figure S7C, Supporting Information). These results were consistent with those of the phosphoproteomic analysis, which also revealed enrichment of the AMPK/mTOR/PPARγ pathway in the FIIN2‐treated groups (Figure S8, Supporting Information).

**Figure 7:**
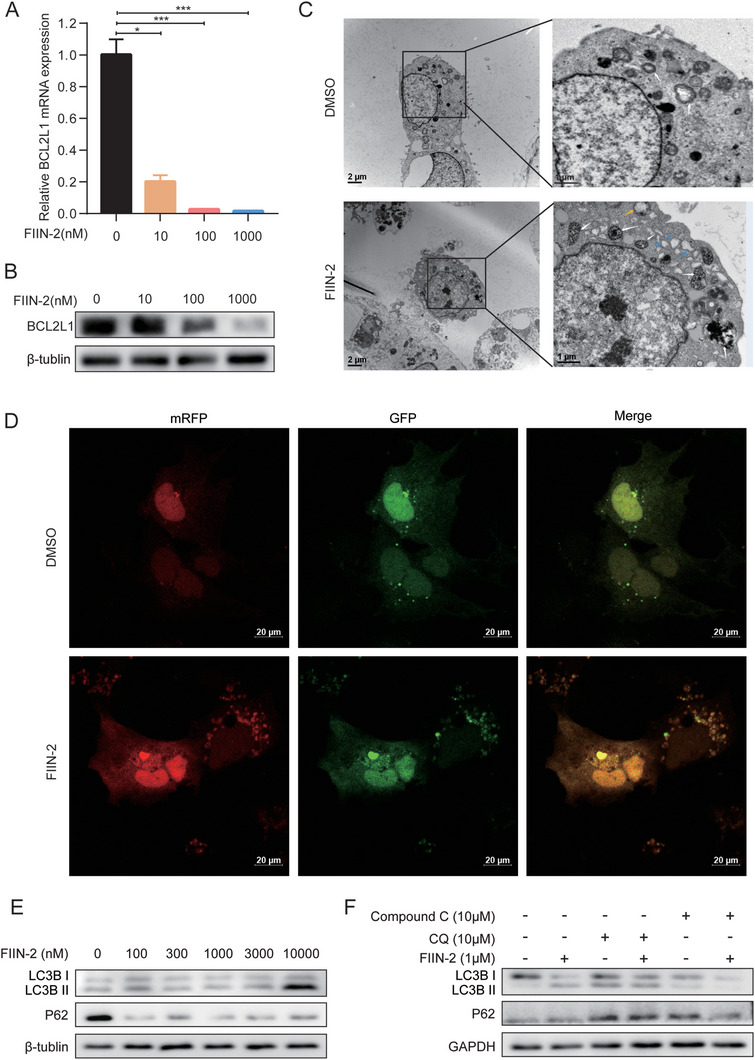
FIIN2 can induce HCC cell autophagy through the AMPK pathway. A) FIIN‐2 downregulated the mRNA expression of BCL2L1. B) FIIN‐2 downregulated the protein expression of BCL2L1. C) Hep3B cells were treated with 1 µm FIIN2 for 16 h, and autophagosomes were detected via transmission electron microscopy (TEM). D) Representative images of mRFP‐GFP‐LC3 puncta in Hep3B cells treated with 1 µm FIIN2 or DMSO. Scale bar: 20 µm. E) LC3‐II/LC3‐I and p62 were detected by western blot in Hep3B cells treated with different concentrations of FIIN2. (F) LC3‐II/LC3‐I and p62 were detected by western blot in Hep3B cells treated with FIIN2, CQ, or Compound C.

Furthermore, combined transcriptomics and proteomics analysis revealed decreased mRNA and protein expression of Bcl‐2‐like protein 1 (BCL2L1), which may be regulated by the transcription factor PPARγ (Figure 5C). The decrease in BCL2L1 induced by FIIN2 was further verified by qPCR and western blotting (**Figure** **7**A,B). AMPKɑ1 overexpression promoted the downregulation of BCL2L1 and other downstream proteins, such as PIP4P2 and SERPINE1, by FIIN2 (Figure S7B, Supporting Information). These results suggest that FIIN2 may exert unexpected functions in HCC by activating the AMPK/mTOR/PPARγ pathway to regulate BCL2L1.

Since both AMPK and BCL2L1 are important in autophagy, we wondered whether FIIN‐2 could induce autophagy in HCC cells. Transmission electron microscopy (TEM) revealed that FIIN‐2 promoted more autophagosome formation (Figure 7C). Additionally, FIIN‐2 distinctly increased autophagic flux in Hep3B cells after mRFP‐GFP‐LC3 lentiviral vector transfection (Figure 7D). FIIN‐2 could also increase the level of LC3‐II and decrease the expression of p62 in Hep3B cells (Figure 7E), while the overexpression of AMPKɑ1 enhanced this effect (Figure S7B, Supporting Information); the induction of LC3‐II by FIIN‐2 can be antagonized by Compound C, which is an inhibitor of AMPK (Figure 7F). These results revealed that FIIN‐2 could bind AMPKα1 at Cys185 to activate the AMPK signaling pathway and induce HCC cell autophagy.

### 2.6 Integrated Multi‐Omics Analysis Reveals the Regulatory Network of FIIN‐2

**Figure 8:**
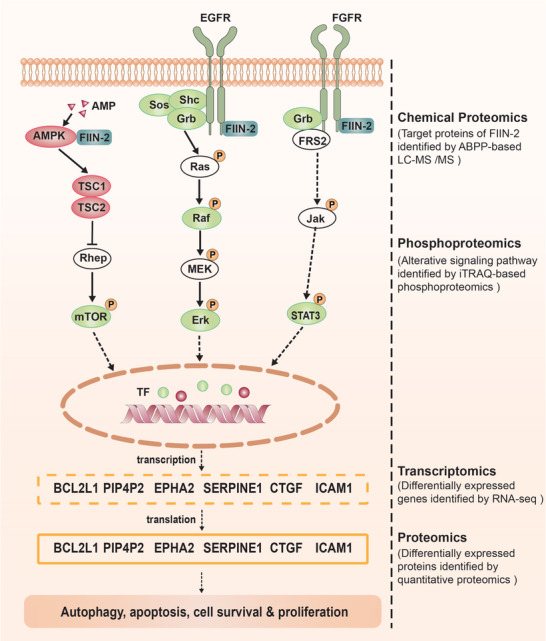
The proposed model integrating multi‐omics data of FIIN‐2 highlights the regulatory network of downstream and involved signaling pathways. The proteins in the green ellipse are downregulated in phosphoproteomics, the proteins in the red ellipse are upregulated in phosphoproteomics, and the proteins in the white ellipse are not altered in phosphoproteomics. ABPP, activity‐based protein profiling; TF, transcription factor.

To obtain a comprehensive understanding of the mechanism of FIIN‐2 in HCC, we combined chemical proteomics with phosphoproteomics, transcriptomics, and proteomics and constructed a relatively complete regulatory network of FIIN‐2 (**Figure** **8**). In the regulatory network, upon binding to target proteins, including FGFRs, EGFR, and AMPKα1, FIIN‐2 sequentially inactivates or activates related signaling pathways by altering the phosphorylation levels of key proteins in the corresponding signaling pathways. For example, the phosphorylation of growth factor receptor‐bound protein (Grb) and STAT3 in the FGFR signaling pathway; Sos, Src homology 2 domain‐containing transforming protein (Shc), and Grb in the EGFR signaling pathway; and mTOR and S6K in the AMPK signaling pathway is decreased, whereas the phosphorylation of tuberous sclerosis complex 1 (TSC1) and TSC2 in the AMPK signaling pathway is elevated. The altered signaling pathways suppress the expression of correlated transcription factors, such as STAT3, Jun, and PPARγ, and ultimately induce decreased expression of downstream effectors, including BCL2L1, SERPINE1, PIP4P2, EPHA2, CTGF, and ICAM1. Finally, the proteins affected by FIIN‐2 function during autophagy, apoptosis, survival, and proliferation, ultimately affecting HCC progression. In summary, an unbiased investigation of chemical proteomics, phosphoproteomics, transcriptomics, and proteomics data that are differentially modulated by FIIN‐2 revealed that FGFR‐targeted drugs may have multiple targets that can exert various biological functions through multiple pathways. These results provide a comprehensive perspective to elucidate the mechanism of FGFR‐targeted inhibitors.

## 3 Discussion

In this study, we used a chemical proteomics approach, which employed an activity‐based probe containing a clickable alkyne and reserving nearly the same biological function as FIIN‐2, to systematically identify the target protein profile of FIIN‐2. We then utilized multi‐dimensional integration analysis and validation, including phosphoproteomics, proteomics, and transcriptomics, to elucidate the potential role and mechanism of FIIN2 by directly targeting these proteins.

We found that FIIN‐2 binds to multiple targets, including kinases and nonkinases. For the kinases, in addition to the known targets (FGFRs, EGFR, SRC),^[^ ^16^ ^]^ AMPKα1 was unexpectedly identified as a novel target of FIIN‐2. We preliminarily determined that FIIN‐2 binds to the Cys185 site of AMPKα1 via a different mechanism. In contrast to FGFR and EGFR, which possess transmembrane catalytic domains, AMPKα1 is an intracellular protein kinase that belongs to different kinase families. As an important energy sensor, AMPK plays a key role in regulating cellular energy metabolism. Through direct phosphorylation of TSC and Raptor, AMPK can inhibit mTOR activity, which in turn inhibits protein synthesis and cell proliferation by inhibiting the kinases eukaryotic translation initiation factor 4E‐binding protein 1 (4eBP1) and ribosomal protein S6 (S6), respectively. AMPK may also indirectly inhibit the Hippo pathway through phosphorylation of hydroxymethylglutaryl‐CoA reductase (HMGR), thereby reducing cholesterol synthesis.^[^ ^17^ ^]^ Thus, AMPK could act as a potential therapeutic target in some dysregulated metabolic diseases, such as diabetes and obesity.^[^ ^18^ ^]^ In recent years, the role of AMPK in tumors has gained increasing attention. For example, recent studies revealed that FIIN‐2 can induce autophagy in lung adenocarcinoma cells through the inhibition of mTOR phosphorylation.^[^ ^19^ ^]^ We also demonstrated that FIIN‐2 can induce HCC cell autophagy by activating the AMPK signaling pathway. Moreover, our phosphoproteomic study revealed that FIIN‐2 can increase the phosphorylation of AMPK and decrease the phosphorylation of HMGR, mTOR, and its downstream S6 kinase. These results suggest that FIIN‐2 might affect HCC progression partly by directly binding to and activating AMPKα1, thereby participating in important biological processes, such as lipid metabolism, cell proliferation, and autophagy, and ultimately influencing the progression of HCC. However, AMPK is a double‐edged sword in tumors,^[^ ^20^ ^]^ and the effect of AMPK on the antitumor activity of FIIN‐2 needs to be thoroughly and comprehensively investigated rather than simply summarized.

Additionally, some potential nonkinases were also identified via chemical proteomics to be targeted by FIIN‐2. Those nonkinases were enriched mainly in metabolic pathways, fatty acid metabolism, protein processing in the endoplasmic reticulum, etc. For example, cytochrome P450 family 2 subfamily J member 2 (CYP2J2), trans‐2,3‐enoyl‐CoA reductase (TECR), and ELOVL fatty acid elongase 1 (ELOVL1) were identified as the top 10 potential target proteins of FIIN‐2 by chemical proteomics, and those three nonkinase proteins were involved in metabolic processes, including lipid or fatty acid metabolism.^[^ ^21^ ^]^ Recent studies revealed that infigratinib, an FGFR inhibitor that was approved by the FDA for the treatment of advanced or metastatic cholangiocarcinoma, could lead to potent reversible inhibition and inactivation of CYP2J2.^[^ ^22^ ^]^ CYP2J2 is expressed predominantly in the heart, and many clinical studies have implied that inactivation of CYP2J2 is related to deleterious cardiac electrophysiological and cardiotoxic effects.^[^ ^23^ ^]^ Thus, we speculated that CYP2J2 could be an off‐target of FIIN‐2 and might induce unexpected side effects. Similarly, other nonkinases may act as on‐targets or off‐targets of FIIN‐2 to regulate various signaling pathways, thereby affecting drug efficacy.

In addition to chemical proteomics and phosphoproteomics, we also acquired gene expression profiles and protein profiles altered by FIIN‐2 through transcriptomics and proteomics. Upon binding and inhibiting EGFR, FIIN‐2 can regulate various genes, such as EphA2,^[^ ^24^ ^]^ CHPF.^[^ ^25^ ^]^ and solute carrier family 38 member 2 (SLC38A2),^[^ ^26^ ^]^ to restrict cancer cell proliferation, invasion, metastasis, and apoptosis through the Ras‐Raf‐MEK‐ERK signaling network. FIIN‐2 can target FGFR to regulate genes such as CTGF,^[^ ^27^ ^]^ ICAM,^[^ ^28^ ^]^ and MAF bZIP transcription factor F (MAFF).^[^ ^29^ ^]^ through the JAK/STAT3 signaling axis, thus participating in tumorigenesis, the tumor microenvironment, and drug resistance. AMPK activated by FIIN‐2 might regulate BCL2L1,^[^ ^30^ ^]^ SERPINE1,^[^ ^31^ ^]^ PIP4P2,^[^ ^32^ ^]^ or other potential genes via the TSC‐mTOR pathway to influence cell apoptosis, lipid metabolism, the immune response, and tumor malignancy. Specifically, BCL2L1, an anti‐apoptotic protein, can inhibit autophagy through binding to Beclin 1, which is necessary to initiate autophagy.^[^ ^33^ ^]^ Moreover, BCL2L1 can be upregulated by the activation of peroxisome proliferator‐activated receptor gamma (PPARγ),^[^ ^34^ ^]^ which is a transcription factor that belongs to the nuclear hormone receptor superfamily.^[^ ^35^ ^]^ Our study revealed that FIIN2 could downregulate the mRNA and protein expression of BCL2L1 and induce autophagy. Moreover, our combined analysis suggested that AMPK could downregulate PPARγ by inhibiting the activity of mTOR.^[^ ^36^ ^]^ Therefore, our integrative analysis revealed that FIIN2 might target AMPK and inhibit the mTOR‒PPARγ pathway to regulate BCL2L1, thus impacting diverse important biological processes, such as apoptosis and autophagy. In addition, mRNA and protein expression were negatively correlated according to the results of the transcriptome and proteome correlation analyses. In fact, many factors may cause mRNA and protein expression to be negatively correlated. Gene transcription is regulated by chemical modifications, while mRNAs themselves may be modified by methylation, regulating their stability and translation efficiency. Protein stability is another factor. Some proteins have long half‐lives, and post‐translational modifications can also regulate protein stability, which confounds and results in a negative correlation between mRNA and protein expression.

From the multi‐omics integration analysis, we systematically compiled a complete regulatory network of FIIN‐2 from directly targeted kinases or nonkinases, their downstream signaling pathways to regulatory transcription factors, the corresponding final effect proteins, and their consequent cell biological processes. We revealed that the tumor suppressive effect of FIIN‐2 was implemented not only by targeting FGFR but also by targeting AMPKα1, EGFR, and other unknown proteins in a synergistic or antagonistic manner. For example, activation of AMPK can overcome the anti‐EGFR antibody resistance induced by KRAS proto‐oncogene mutations in colorectal cancer, while high levels of AMPK activity can lead to apoptosis when colorectal cancer cells are treated with wild‐type KRAS and anti‐EGFR antibodies.^[^ ^37^ ^]^ This phenomenon was also observed between AMPK and FGFR. In gastric cancer patients resistant to FGFR inhibitors, activation of transforming growth factor‐β‐activated kinase 1 (TAK1) can induce autophagy in an AMPK‐dependent manner.^[^ ^38^ ^]^ Therefore, simultaneous targeting of these two kinases may have a synergistic antitumor effect. Our results showed that FIIN‐2 can target both kinases simultaneously. On the one hand, it can inhibit the phosphorylation of EGFR and its downstream signaling. On the other hand, it can activate AMPK and inhibit the mTOR pathway, thus participating in various biological processes and influencing the antitumor effect of FIIN‐2. These proteins may regulate common or distinct signaling pathways, forming an intricate network and ultimately contributing to the combined role of FIIN‐2 in HCC. However, since our study aimed mainly to explore the proteome‐wide binding profile of FIIN‐2, provide insights into the mechanism of FIIN‐2, and offer a paradigm for similar studies, there are still more efforts to rigorously demonstrate the impact of these potential targets on the antitumor activity of FIIN‐2.

## 4 Conclusion

In summary, we propose that FIIN‐2 can target multiple kinases and nonkinases, mainly EGFR, FGFR, and AMPKα1, to exert synthetic antitumor effects. These findings greatly increase our understanding of the on‐ and off‐target activities of FIIN‐2, providing a comprehensive view of its molecular interactions in cancer cells. The identification of new targets is beneficial for maximizing drug efficacy, preventing drug side effects, and discovering new drug indications. FIIN‐2 can serve as a lead compound and lay the foundation for the development of more effective drugs by further optimizing drug efficacy and pharmacokinetic properties. Our study underscores the value of integrative multi‐omics strategies for uncovering detailed selectivity profiles and mechanistic insights into covalent kinase inhibitors.

## 5 Experimental Section

### Cell Culture

Hep3B and Huh7 cells were obtained from the Cell Bank of the Chinese Academy of Sciences and were cultured in Minimum Essential Medium (MEM) and Dulbecco's modified Eagle's medium (DMEM) supplemented with 10% FBS. All the cells were grown in a humidified incubator with 5% CO_2_ at 37 °C.

### Cell Viability Assay

Cell viability assays were performed by using a Cell Counting Kit‐8 (CCK8) (Dojindo, Kumamoto, Japan) according to the manufacturer's instructions. The cells were seeded into 96‐well plates at 3 × 10^3^ cells per well in 100 µL of medium and treated with a series of concentrations of FIIN‐2 or FP after attachment. Upon treatment for 72 h, another 10 µL of CCK8 was added to the cells. After incubation for 1 h, the absorbance values of the cells were detected at 450 nm with a PowerWave XS Microplate Reader (BIO‐TEK). The inhibition rate was calculated via GraphPad Prism 7.

### Western Blotting

The cells were harvested and lysed in lysis buffer (20 mm Tris‐HCl, pH 7.5; 150 mm NaCl; and 1% Triton X‐100) supplemented with protease and phosphatase inhibitors. An equal amount of protein lysate from each sample was separated on 6–10% SDS‒PAGE gels and transferred to PVDF membranes following standard operating instructions. After being blocked with 5% nonfat milk, the membranes were incubated with primary antibodies overnight at 4 °C. The primary antibodies used in this study were as follows: FGFR4 (Cell Signaling Technology, 8562), pFGFR (Tyr653/654) (Cell Signaling Technology, 3476), MAPK (Cell Signaling Technology, 9107), pMAPK (Thr202/Tyr204) (Cell Signaling Technology, 9101), AKT (Cell Signaling Technology, 4691), pAKT (Ser473) (Cell Signaling Technology, 4060), EGFR (Cell Signaling Technology, 4267), pEGFR (Thr693) (Cell Signaling Technology, 3056), AMPKα1 (Cell Signaling Technology, 2795), pAMPK (Thr172) (Cell Signaling Technology, 2535), mTOR (Immunoway Biotechnology, YT2913), p‐mTOR (Ser2448) (Immunoway Biotechnology, YP0176), FGFR2 (Cell Signaling Technology, 11835), Src (Cell Signaling Technology, 2109), LC3 (Proteintech, 14600‐1‐AP), P62 (Proteintech, 18420‐1‐AP), CTGF (Proteintech, 25474‐1‐AP), ICAM1 (Proteintech, 10831‐1‐AP), EPHA2 (Cell Signaling Technology, 6997), CHPF (Santa Cruz Biotechnology, sc‐376183), PIP4P2 (Immunoway, YT7377), and SERPINE1 (Proteintech, 13801‐1‐AP), GAPDH (Proteintech, 10494‐1‐AP), β‐tublin (Proteintech, 10094‐1‐AP).

### Gel‐Based ABPP

The cells were cultured in 6‐well plates and treated with DMSO, FP, or FP pretreatment with FIIN‐2 at 37 °C for 1 h. Gel‐based ABPP was performed on the basis of previously reported procedures.^[^ ^39^ ^]^ After the medium was removed, the cells were washed with ice‐cold Dulbecco's phosphate‐buffered saline (DPBS) twice, lysed in lysis buffer containing 0.1% SDS, 50 mm Tris‐HCl, 150 mm NaCl, and protease inhibitor cocktail (Roche) for 30 min and sonicated for 2 min. The cell lysate was collected by centrifugation at 120 00 rpm for 30 min at 4 °C, and the protein concentration was determined via a BCA assay (Thermo Fisher Scientific). Click reaction was conducted at room temperature (RT) for 1 h in a final volume of 50 µL, including 43 µL of protein (1 mg mL^−1^), 100 µm Tetramethylrhodamine(TAMRA)‐azide (Sigma–Aldrich), 1 mm Tris(2‐carboxyethyl)phosphine (TCEP, Sigma–Aldrich), 100 µm Tris[(1‐benzyl‐1H‐1,2,3‐triazol‐4‐yl)methyl]amine (TBTA, Sigma–Aldrich), and 1 mm CuSO4 (Sigma–Aldrich). The reaction was subsequently quenched by the addition of 12.5 µL of loading buffer. 30 µL of each sample was loaded and separated on a 10% SDS‒PAGE gel and visualized by in‐gel fluorescence using a flatbed scanner (Typhoon, GE).

To label the recombinant protein by FP, AMPKɑ1 (WT or C185A mutant, 0–20 µg) was incubated with FP (10 µm) at 25 °C for 20 min. Each sample was added to a premixture with a final concentration of 50 µm TAMRA‐azide, 1 mm TCEP, 100 µm TBTA, and 1 mm CuSO4 supplemented with buffer to a volume of 25 µL. The reactions were carried out at RT for 45 min in the dark and subsequently terminated by the addition of 6.25 µL of 5 × loading buffer and heating at 95 °C for 5 min. 20 µL of each sample was loaded and separated on a 10% SDS‒PAGE gel and visualized by in‐gel fluorescence using a flatbed scanner (Typhoon, GE). After fluorescence scanning, the proteins were stained with Colloidal Blue (Invitrogen).

### Chemical Proteomics—Click Reaction and Target Protein Enrichment

The cells cultured in 10‐cm plates were treated with DMSO, FP, or FP pretreatment with FIIN‐2. After the medium was aspirated, the cells were washed with DPBS twice, lysed in 750 µL of lysis buffer (0.2% SDS, 0.1% IGEPAL CA‐630, and PBS) containing protease inhibitor cocktail (Roche) and sonicated for 15 min at 4 °C. The lysate was centrifuged at 20 000 × *g* for 30 min at 4 °C, and the supernatant was transferred into a new tube. The concentration of the supernatant was examined via the Bradford assay, and the lysate was subsequently diluted to 2 mg mL^−1^ with PBS (plus protease inhibitors). Click chemistry was performed on each sample with 900 µL of protein lysate, 4 µL of biotin‐PEG3‐azide (5 mm), 40 µL of TBTA/CuSO4 (25 mm/12.5 mm), and 40 µL of ascorbate sodium (250 mm). The sample was gently vortexed after each addition, and the reaction was allowed to proceed at room temperature for 1 h in the dark, followed by the addition of an 8 fold volume of CHCl_3_‐MeOH‐H_2_O (4:1:3) to precipitate the proteins. The precipitated proteins were washed twice with 500 µL of ice‐cold methanol and centrifuged at 14 000 × *g* for 5 min at 4 °C. Air‐dried protein pellets were resuspended via sonication in 300 µL of 1.2% SDS/PBS. After cooling to RT, possible precipitated impurities were removed by centrifugation at 20 000 × *g* for 2 min, and the solution was diluted with PBS to 0.2% SDS/PBS. Then, 100 µL of prewashed streptavidin beads was added to each sample and incubated on a rotator for 3 h at RT. After enrichment, the beads were washed with 1.8 mL of 0.2% SDS/PBS and mixed at RT for 10 min. The beads were transferred to a new tube and washed six times with PBS. Then, 500 µL of resuspension buffer (6 m urea/100 mm triethylammonium bicarbonate [TEAB]) was added to the beads with an additional 10 mm dithiothreitol (DTT) and rotated at 37 °C for 30 min. Subsequently, 20 mM iodoacetamide (IAA) was added, and the mixture was incubated for 30 min in the dark at RT. The resin was washed with 1 mL of 200 mm 3‐[4‐(2 ‐Hydroxyethyl) piperazin‐1‐yl] propane‐1‐sulfonic acid (EPPS) three times, pH 8.5. The resin was resuspended in 50 µL of 200 mm EPPS, and an additional 2 µL of LysC (0.5 g L^−1^) was added and incubated overnight at 37 °C. Further digestion was performed by adding 2 µL of trypsin (0.5 g L^−1^) to the mixture and rotation incubation for 6 h.

### Chemical Proteomics—TMT Labeling and LC/MS‐MS Analysis

The digested peptides were centrifuged at 20 000 × *g* for 2 min, and the supernatant was transferred to a new tube. The peptides of each sample were labeled with TMT reagent and separated by high‐pH high‐performance liquid chromatography (HPLC). Mobile phase A (2% acetonitrile (ACN)‐98% H_2_O, adjusted to pH 10.0 with NH3·H_2_O) and mobile phase B (98% ACN‐2% H_2_O, adjusted to pH 10.0 with NH3·H_2_O) were used for peptide elution. The gradient separation conditions were as follows: 5% B in 0–6 min; 5–8% B in 7–8 min; 8–19% B in 9–19 min; 19–32% B in 20–28 min; 95% B in 29–31 min; 95–15% B in 32–33 min; and 15% B in 34 min. Fractionation was performed at a flow rate of 1.5 mL min^−1^. The eluate was collected every min and combined into 6 fractions before freeze‐drying by vacuum centrifugation. The spin‐dried peptides were redissolved in 0.1% (v/v) formic acid (FA) solution and then subjected to an Easy‐nLC 1200 LC system (Thermo Fisher Scientific) coupled with a Q Exactive HF‐X Orbitrap mass spectrometer (Thermo Fisher Scientific) for LC‐MS/MS analysis. The peptides were separated on an Easy‐nLC 1200 UPLC system with an analytical column (25 cm, 75 µm id.) at a flow rate of 300 nL min^−1^; buffer A, 0.1% FA in water; and buffer B, 0.1% FA in acetonitrile (ACN). The gradient was set as follows: 0–5 s, 3–5% buffer B; 6 s‐40 min, 5–15% buffer B; 41–75 min, 15–28% buffer B; 76–87 min, 28–38% buffer B; and within 5 s, buffer B was increased to 100% and maintained for 8 min. The fractioned peptides were ionized under 2 kilovolts. The MS1 full scan was set at a resolution of 60 000, and the ions with m/z ranged from 350 to 1800 Da. The MS2 spectrum was operated in up to 20 data‐dependent acquisition modes by high‐energy collision‐induced dissociation (HCD) (normalized collision energy of 28%) at a resolution of 15 000. The maximal time for ion injection was 50 ms, and the maximal time for dynamic exclusion was 30 s. The raw mass spectrometric files were searched against the human UniProt database via MaxQuant (version 1.6.5.0).^[^ ^40^ ^]^

### In Situ Pulldown and Target Protein Validation

For the in situ pulldown assay, cells were treated and lysed as described for gel‐based ABPP. The protein concentration of each sample was normalized to 2 mg mL^−1^. The click reaction was performed at RT for 1 h in a final volume of 500 µL, including 430 µL of protein mixture (2 mg mL^−1^), 100 µm biotin‐azide (Sigma–Aldrich), 1 mm TCEP, 100 µm TBTA and 1 mm CuSO4. Then, the proteins were precipitated by adding 2 mL of prechilled MeOH, 0.5 mL of CHCl_3_, and 1.5 mL of H_2_O overnight at −20 °C. The precipitated proteins were collected by centrifugation at 7000 rpm for 20 min at 4 °C, and the supernatant was discarded. The residue was washed twice by resuspending the pellet in 1 mL of MeOH and centrifuging at 12 000 rpm for 10 min. The protein pellet was resuspended with sonication in 2 mL of 0.2% SDS/PBS, and 100 µL of prewash streptavidin beads were added. The mixture was incubated on a rotator for 3 h at RT. The beads were washed with 1% SDS/PBS and PBS containing 300 mm NaCl three times at RT. The beads were collected by centrifugation at 2000 rpm for 3 min, and 200 µL of sodium dithionite buffer (50 mm Na_2_S_2_O_4_ in 1% SDS/PBS) was added for rocking at RT for 30 min to elute the enriched proteins. The eluted proteins were precipitated with ice‐cold MeOH and collected by centrifugation at 12 000 rpm for 10 min. Finally, 20 µL of 2 × loading buffer was added to the protein pellets, and all the samples were subjected to SDS‒PAGE separation. A total of 20 µg of whole‐cell lysate was loaded as input for comparison. Subsequent immunoblotting was performed as described above.

### Immunofluorescence

Hep3B cells were treated with FP (10 µm) or DMSO for 1 h. The cells were fixed with 4% polyformaldehyde for 30 min, and then 0.1% Triton X‐100 was added for 5 min. For the click reaction, a premixture with a final concentration of 50 µm TAMRA‐azide, 1 mm TCEP, 100 µm TBTA, and 1 mm CuSO4 was added, and the cells were gently rotated at RT for 1 h. The cells were blocked with 5% BSA for 1 h at RT, further incubated with primary antibody at 4 °C overnight, and then with goat anti‐rabbit IgG H&L (Alexa Fluor 488) secondary antibody for 1 h at RT. All the samples were washed twice with PBS, followed by staining with DAPI (Solarbio) prior to imaging. Imaging was performed with a ZEISS LSM 900 with Airyscan 2.

### Computational Modeling

The protein structure used for modeling FIIN‐2 with AMPKɑ1 (PDB:6C9J) was obtained from the Protein Data Bank (http://www.rcsb. org/pdb). The proteins and compounds were prepared via the Protein Preparation Wizard and LigPrep in Maestro, respectively. Covalent docking was performed with default settings via the CovDock workflow in the Schrödinger suite and then plotted in PyMOL.

### Protein Expression and Purification

The kinases AMPKɑ1 (residues 1‐312) and the mutant (C185A) were cloned and inserted into a modified pET28a expression vector with an N‐terminal 6 × His tag. The plasmid was transformed into DE3‐competent *E. coli* cells, and the transformants were selected on LB agar plates containing kanamycin. For large‐scale protein expression, 1 L of LB broth was inoculated with 10 mL of overnight culture and grown in an incubator at 37 °C until an OD600 of 0.6–0.8 was reached. Protein expression was induced for 18 h with 0.5 mm isopropyl‐β‐D‐thiogalactopyranoside (IPTG) at 18 °C. The cells were pelleted via centrifugation and resuspended in lysis buffer (50 mm Tris, pH 7.4; 150 mm NaCl; 10% glycerol; 2 mm TCEP; and 20 mm imidazole). The cell suspension was sonicated on ice for 7 min at a 25% duty cycle. The insoluble material was removed by centrifugation at 15 000 rpm for 30 min at 4 °C, and the resulting supernatant was used for subsequent protein purification. Protein purification was conducted at 4 °C by Ni‐NTA affinity chromatography, and bound proteins were eluted with elution buffer (lysis buffer containing 300 mm imidazole). The purified proteins were concentrated and stored at −80 °C for subsequent studies.

### Microscale Thermophoresis (MST)

Purified 6 × His‐tagged AMPKɑ1 WT or C185A was labeled with RED‐Tris‐NTA 2nd Generation dye (NanoTemper Technology, MO‐L018) according to the manufacturer's instructions. FIIN2 was diluted in various concentrations and then mixed with 200 nm labeled AMPKɑ1 WT or C185A in PBS (supplemented with 200 mm NaCl). The MST assays were carried out on a Monolith NT.115 instrument (NanoTemper Technology). The thermophoresis data were recorded in expert mode with the default parameters provided by MO. Control program. Fitting curves and KD values were generated via MO. Affinity Assay software.

### Kinase Assay

The ADP‐GloTM Kinase Assay Kit (Part No #V9101) was purchased from Promega. The experiments were performed according to the manufacturer's instructions. AMPK activity was determined by phosphorylation of the SAMS peptide (GenScrit, sequence: HMRSAMSGLHLVKRR) and the generation of ADP. First, purified AMPKɑ1 WT or C185A (2 µm) was incubated with a mixture containing 100 µm SAMS peptide, 50 µm ATP (Promega), 20 mm MgCl_2_, 100 µm FIIN2 and kinase assay buffer (50 mm Tris pH 7.4, 20 mm NaCl, 1 mm DTT, 4% DMSO, 0.01% BSA and 0.02% (v/v) Tween‐20). Then, ADP‐Glo Reagent was added to terminate the kinase reaction and deplete the remaining ATP. Finally, the kinase detection reagent was added to convert ADP to ATP, and the newly synthesized ATP was measured on a multimode plate reader (Perkin–Elmer).

### Transmission Electron Microscopy (TEM)

Hep3B cells were treated with FIIN2 (1 µm, 16 h), and DMSO‐treated samples were used as controls. The cells were collected and centrifuged at 1000 rpm for 5 min. The cells were fixed with 2.5% glutaraldehyde at 4 °C overnight and postfixed with 2% osmium tetroxide for 2 h. Next, the cells were dehydrated with cold‐graded ethanol and then rinsed with acetone. The cells were subsequently embedded in the SPI‐Pon 812 medium. Sections (90 nm) were cut and stained with 0.2% lead citrate and 1% uranyl acetate. Images were acquired via transmission electron microscopy (H‐7650, Hitachi, Japan).

### Autophagic Flux Analysis

Autophagic flux was analyzed via the mRFP‐GFP‐LC3 vector. The cells were seeded in 6‐well plates and transfected with the mRFP‐GFP‐LC3 vector, followed by treatment with 1 µm FIIN‐2 or DMSO for 24 h. After being fixed with 4% paraformaldehyde and washed with PBS three times, the cells were imaged via a confocal microscope.

### Proteomic and Phosphoproteomic Analysis—Protein Isolation and Digestion

The collected cells were transferred into 1.5 mL Eppendorf tubes, and an appropriate amount of precooled cell lysate and a final concentration of 1 × cocktail containing EDTA and phosphatase inhibitor were added and mixed thoroughly. After 5 min on ice, a final concentration of 10 mm DTT was added. The samples were sonicated for 2 min on ice. The extracts were clarified by centrifugation at 4 °C for 15 min at 25 000 × *g*. The protein concentrations were determined via a BCA assay (Thermo Fisher Scientific). Protein samples were reduced with 10 mm DTT and alkylated with 55 mm iodoacetamide before trypsin digestion at a 1/20 enzyme/protein ratio at 37 °C for 4 h. Finally, the samples were desalted with a C18 column and lyophilized.

### Proteomic and Phosphoproteomic Analysis—iTRAQ Labeling

One hundred micrograms of each digested sample was added to 100 µL of 0.5 m TEAB buffer. Isobaric labeling of the samples was performed via iTRAQ reagents (Thermo Fisher Scientific). The samples were labeled according to the iTRAQ reagent kit instructions. Briefly, iTRAQ reagents were brought to room temperature and dissolved in isopropanol. The peptides were labeled via the addition of an iTRAQ tag. The labeling reagents were incubated for 2 h at room temperature. The reactions were stopped by adding 100 µL of Milli‐Q water. Finally, the peptides were desalted with a C18 column and lyophilized. These experiments were performed in triplicate.

### Proteomic and Phosphoproteomic Analysis—Phosphopeptide Enrichment

Phosphopeptides were enriched via the High‐Select TiO_2_ Phosphopeptide Enrichment Kit (Thermo Scientific PN32993) according to the vendor's protocol. Briefly, dried peptides were redissolved in 150 µL of binding/equilibration buffer and applied to the equilibrated TiO_2_ spin twice. The spin tip was sequentially washed twice with 20 µL of binding buffer and wash buffer and once with 20 µL of LC‒MS‐grade water. The bound peptides were eluted twice with 50 µL of elution buffer. The eluates were lyophilized and subsequently redissolved in 50 µL of 0.1% FA for peptide concentration measurement by measuring the absorbance at 280 nm (NanoDrop 2000, Thermo Scientific).

### Proteomic and Phosphoproteomic Analysis—High‐pH Reversed‐Phase Fractionation

Peptides were fractionated via the Pierce High pH Reversed‐Phase Peptide Fractionation Kit (Thermo Scientific PN84868) following the manufacturer's manual. Briefly, peptides were dissolved in 0.1% TFA buffer and subsequently loaded onto fractionation spin columns. The samples were then washed with 5% ACN/0.1% TFA to remove contaminants. The peptides were eluted into six fractions with an ACN step gradient. The samples were acidified and lyophilized before LC‐MS.

### Proteomic and Phosphoproteomic Analysis—LC‒MS/MS Analysis

For the whole proteome or phosphoproteome, two microliters (1 µg) of each sample were injected onto a 0.075 × 25 cm C18 column attached to an Ultimate 3000 UHPLC (Thermo Fisher Scientific). The mobile phases consisted of 0.1% FA (A) and 2% ACN (B). The peptides were separated via 0.1% FA as solvents at a flow rate of 300 nL per minute with a 1‐h gradient as follows: 5% B (0–5 min), 5–25% B (5–45 min), 25–35% B (45–50 min), 35–80% B (50–52 min), 80% B (52–54 min), and 5% B (54–60 min). Data were acquired in positive ion data‐dependent mode on a Q Exactive HF mass spectrometer (Thermo Fisher Scientific, San Jose, CA) with a resolution of 60 000 (at m/z 200) and a scan range from m/z 350–1500. The other parameters of the MS scan were as follows: automatic gain control (AGC) target of 3e6; maximum injection time of 50 ms; dynamic exclusion of 30 s; MS/MS scan resolution of 15 000; AGC target of 1e5; and collision energy of 30 eV.

### Proteomic and Phosphoproteomic Analysis—Data Analysis

All MS/MS data were analyzed via Proteome Discoverer 1.4 (Thermo Fisher Scientific) integrated with Mascot (version 2.3). The precursor mass tolerance was set to 20 ppm, and the fragment ion tolerance was 0.05 Da while the digestion enzyme trypsin was assumed, allowing up to two missed cleavages. Fixed modifications were set as follows: carbamidomethyl (C), iTRAQ8plex (N‐tern), iTRAQ8plex (K), and variable modifications as follows: oxidation (m), acetyl (protein N‐term), deamidated (NQ), phosphor (ST), phosphor (Y), and iTRAQ8plex (Y). The data were searched against NCBI_Homo_nr. fasta (81064 sequence) with a 1% FDR. Reporter ion abundances were corrected for isotopic impurities on the basis of the manufacturer's specifications. For each peptide, a minimal average reporter signal‐to‐noise threshold of 2 was used. The signal‐to‐noise values were normalized by the sum of the intensities within each iTRAQ channel. Abundance ratios were calculated from the average peptide abundance of biological replicates. Only peptides identified in at least two biological replicates were considered for further analysis. The phosphoproteome data were filtered to include only phosphopeptides with class I phosphorylation site localization (phosphoRS score >0.75). Phosphopeptides containing identical phosphorylation site localizations but differing in methionine oxidation states or peptide missed cleavages were aggregated to generate a single quantitative value per unique phosphorylation site. The phosphorylation sites quantified in peptides with different phosphorylation states (i.e., doubly or singly phosphorylated) were not merged and remained as separate quantified values. Statistical significance was determined by Student's *t*‐test (*p* value ≤ 0.05).

### RNA‐Seq Analysis

Hep3B cells were plated at a density of 5 × 10^6^ cells in a 100 mm dish. The cells were treated with DMSO (as a control) or 1 µm FIIN‐2 for 6 h. The cells were harvested and stored in TRIzol reagent for RNA sequencing. The DNBSEQ platform was used for sequencing, and the results produced 150 bp (PE150) paired‐end reads. The raw sequencing data were preprocessed, and low‐quality and short reads (≤20 bp) were removed. For quality control, the clean reads were aligned to the reference genome via HISAT. The DESeq2 package was used to perform differential gene expression analysis and differentially expressed genes (DEGs) were defined as those with *p* values less than 0.05 and log2(fold change) ≥1.

### Quantitative qPCR

Total RNA was extracted from cells via TRIzol reagent (Invitrogen) and reverse transcribed using the PrimeScript qRT‒PCR Kit (Takara) according to the manufacturer's instructions. Quantitative qPCR was performed with SYBR qPCR Master Mix (Vazyme) on a LightCycler 480 System (Roche). The relative mRNA level was normalized to that of β‐actin and analyzed via the 2^−ΔΔCt^ method. Three independent biological replicates were carried out under strictly controlled experimental conditions. GraphPad Prism 7 was used for the statistical analysis and data were presented as mean ± standard deviation (SD). Statistical significance was determined by Student's *t* test(ns: no significant, ^*^p < 0.05, ^**^p < 0.01, ^***^p < 0.001, ^****^p < 0.0001). The primers used in this study were listed in Table S7 (Supporting Information).

### Bioinformatic Analysis

GO, KEGG, and disease enrichment analyses were performed using the clusterProfiler R package. Items from the GO, KEGG, or disease categories with *p* values < 0.05 were considered significantly enriched. The Pearson correlation coefficients between mRNA and protein expression under inhibitor treatment conditions were calculated by correlating the values of the log fold changes in mRNA and protein expression before and after inhibitor treatment. Transcription factors and their target genes extracted from MSigDB (version 7.5.1) TFT (transcription factor targets) were used to annotate the dataset. The peptides were aligned and extended to a width of 15 amino acids via a custom R script. Enrichment was determined via default settings (significance level 0.05).

### Statistics Analysis

The statistical analysis of all data, which underpin the findings, were detailed in the corresponding experimental sections.

## Conflict of Interest

The authors declare no conflict of interest.

## Author Contributions

Y.F. and D.Z. contributed equally to the work and should be regarded as co‐first authors. Y.C. and M.L. conceived the project and designed the experiments. Y.F. and D.Z. performed the experiments. G.X. designed and synthesized the chemical probe. M.L. analyzed the omics data. X.C., L.Q., M.G., S.Z., and Z.C. provided construction suggestions. Y.F., D.Z., and M.L. wrote the manuscript. All the authors have read and approved the final manuscript.

## Supporting information



Supporting Information

Supplemental Table S1

Supplemental Table S2

Supplemental Table S3

Supplemental Table S4

Supplemental Table S5

Supplemental Table S6

Supplemental Table S7

## Data Availability

The data supporting the findings of this study are available from the corresponding author upon reasonable request. All multi‐omics raw data can be accessed via the following link: https://www.iprox.cn//page/SCV017.html (Accession Number: ipx0004913000).
