# Supplementary material for: Integrative Multi‐Omics Approaches Reveal Selectivity Profiles and Molecular Mechanisms of FIIN‐2, a Covalent FGFR Inhibitor
Source: Adv Sci (Weinh). 2025 Feb 20;12(14):2412578. doi: 10.1002/advs.202412578 (PMC11984845; doi:10.1002/advs.202412578)
Supplement: Supplementary file 1 — Supporting Information [file ADVS-12-2412578-s007.docx]

**Supplementary materials and methods**

**CCK8 cell proliferation assay**

The cells (1 × 10^3^ cells per well) were seeded in 96-well plates. The cells were treated with DMSO, FIIN-2, FP or TAS120 for 6 days. The absorbance values of the cells each day were detected at 450 nm with a PowerWave XS Microplate Reader (BIO-TEK) after they were incubated with fresh medium containing 10% CCK8 (Vazyme, A311-01) for 1 h at 37 °C.

**Colony formation assay**

The cells (1× 10^3^ cells per well) were seeded in 12-well plates. After 10 days of treatment with DMSO, FIIN-2, FP or TAS120, the cells were fixed with 4% polyformaldehyde for 30 min and stained with 1% crystal violet for 20 min. Finally, the colonies in each well were photographed.

**Lentiviral transfection**

GV341 lentivirus particles encoding AMPKɑ1 were purchased from Genechem (Shanghai, China) and used to infect Hep3B cells according to the manufacturer’s instructions. Stably transduced cell lines were screened with 4 μg/mL puromycin.

**LC‒MS/MS**

Tandem MS analysis of the in vitro binding of FIIN-2 and AMPKɑ1. The samples were reduced with 10 mM DTT and alkylated with 55 mM iodoacetamide before trypsin digestion at a 1/50 enzyme/protein ratio at 37 °C overnight. Finally, the samples were desalted with a C18 column and lyophilized.

Two microliters (1 μg) of each sample were injected onto a 0.075 × 25 cm C18 column attached to a Thermo Vanish UHPLC (Thermo Fisher Scientific). The mobile phases consisted of 0.1% FA (A) and 80% ACN (B). The peptides were separated via 0.1% FA as solvents at a flow rate of 300 nl per minute with a 1-h gradient as follows: 5% B (0–5 min), 5–25% B (5–45 min), 25–35% 368 B (45–50 min), 35–80% B (50–52 min), 80% B (52–54 min), and 5% B (54–60 min). Data were acquired in positive ion data-dependent mode on an Exploris 240 mass spectrometer (Thermo Fisher Scientific, San Jose, CA) with a resolution of 60,000 (at m/z 200) and a scan range from m/z 350--1500. The other parameters of the MS scan were as follows: automatic gain control (AGC) target of 3e6; maximum injection time of 20 ms; dynamic exclusion of 30 s; MS/MS scan resolution of 15,000; AGC target of 1e5; and collision energy of 30 eV. All MS/MS data were analyzed via MaxQuant (version 2.1.3). The precursor mass tolerance was set to 20 ppm, and the fragment ion tolerance was 0.05 Da while the digestion enzyme trypsin was assumed, allowing up to two missed cleavages. Fixed modifications were set as follows: carbamidomethyl (C) and variable modifications as follows: oxidation (M), acetyl (protein N-term), and FIIN2 (C). The data were searched against UniProt human fasta plus the expressed AMPKɑ1 sequence with a 1% FDR.

**Supplementary Figures**

**
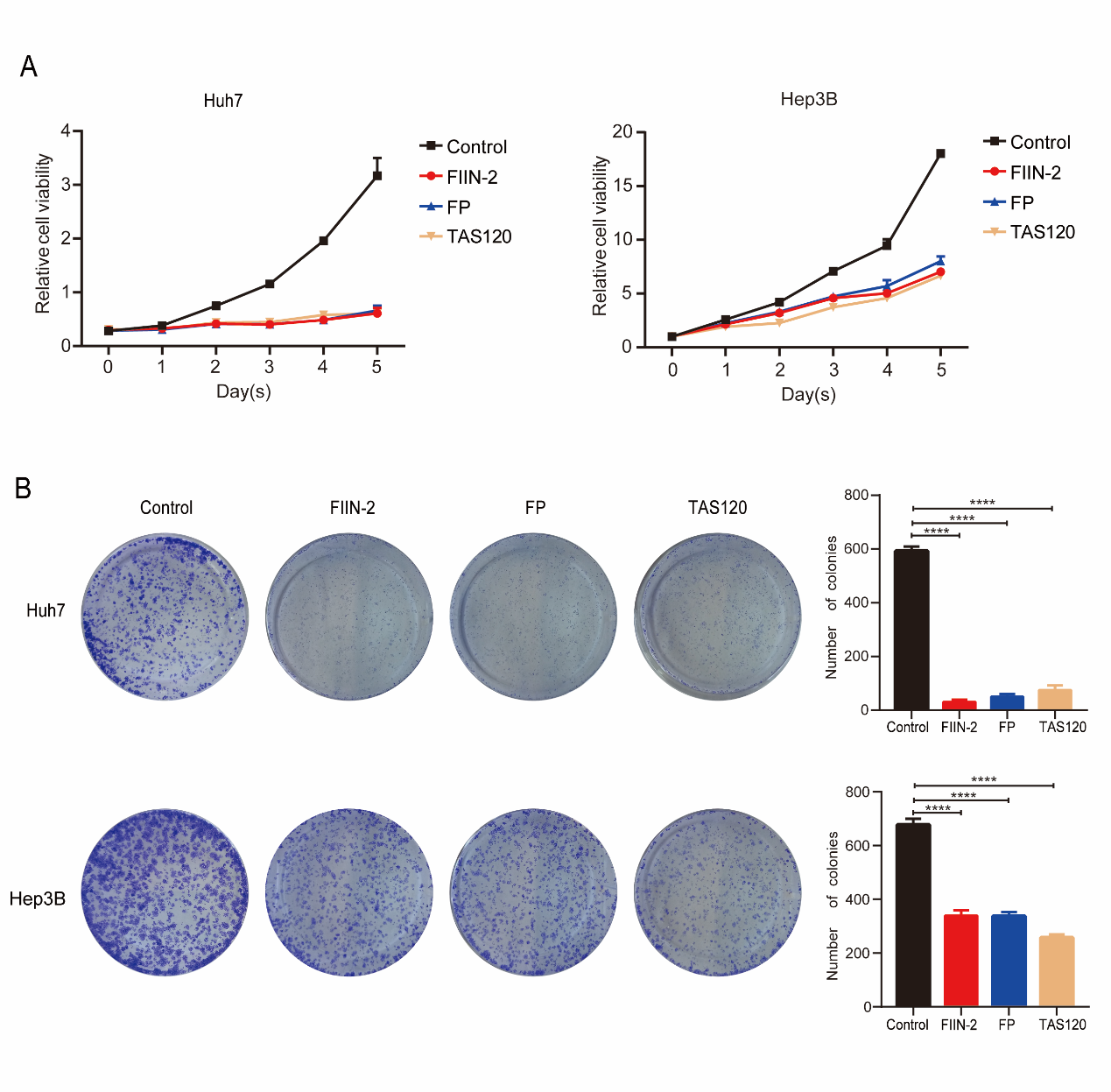
**

**Figure S1. Inhibitory effects of FIIN-2 and FP.** (A) Cell proliferation and (B) colony formation experiments revealed that FIIN-2 exhibited an inhibitory effect similar to that of FP. DMSO and TAS120 were used as negative and positive controls, respectively.

**
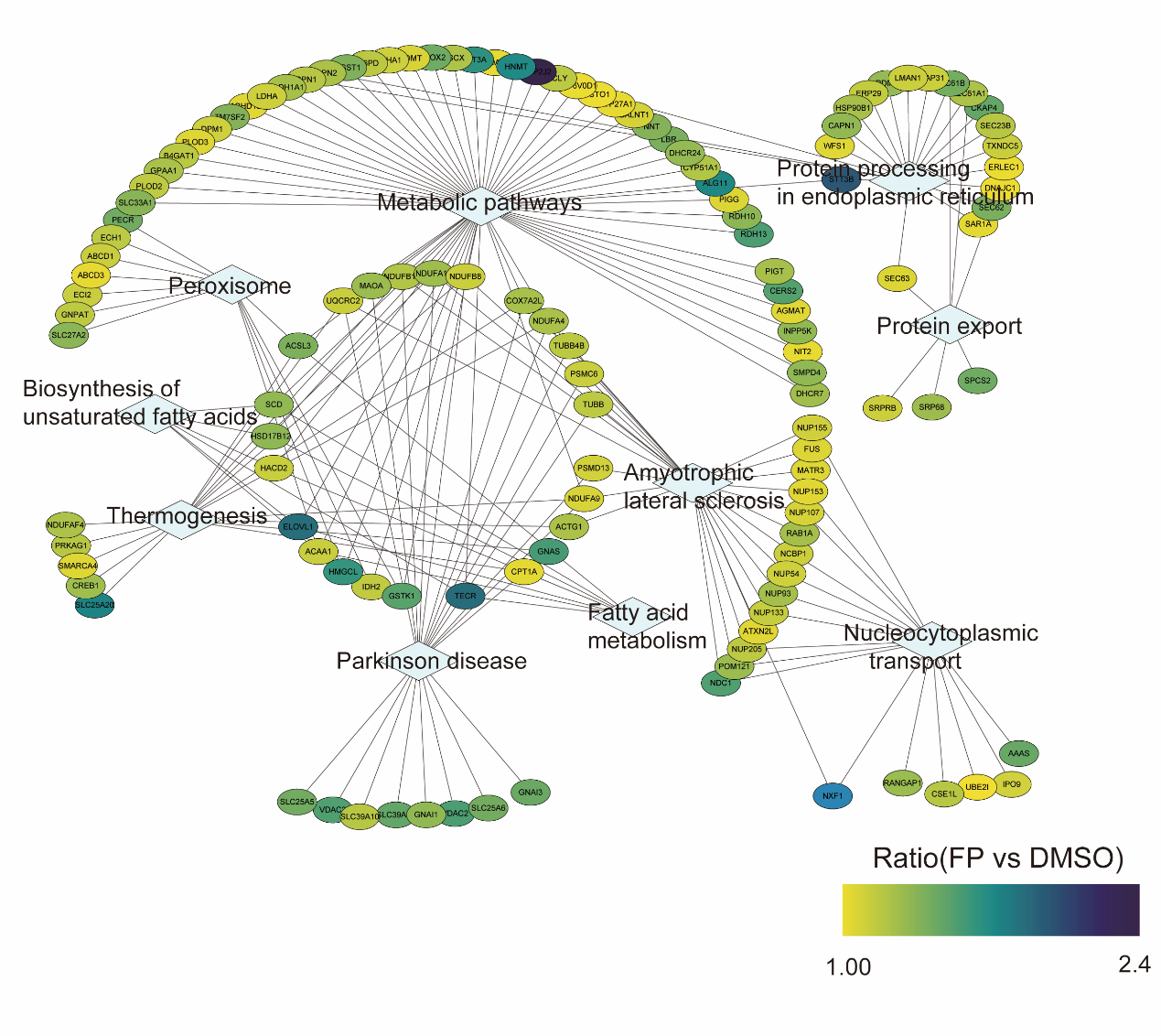
**

**Figure S2.** KEGG enrichment analysis of the non-kinase proteins identified via chemical proteomics.

**
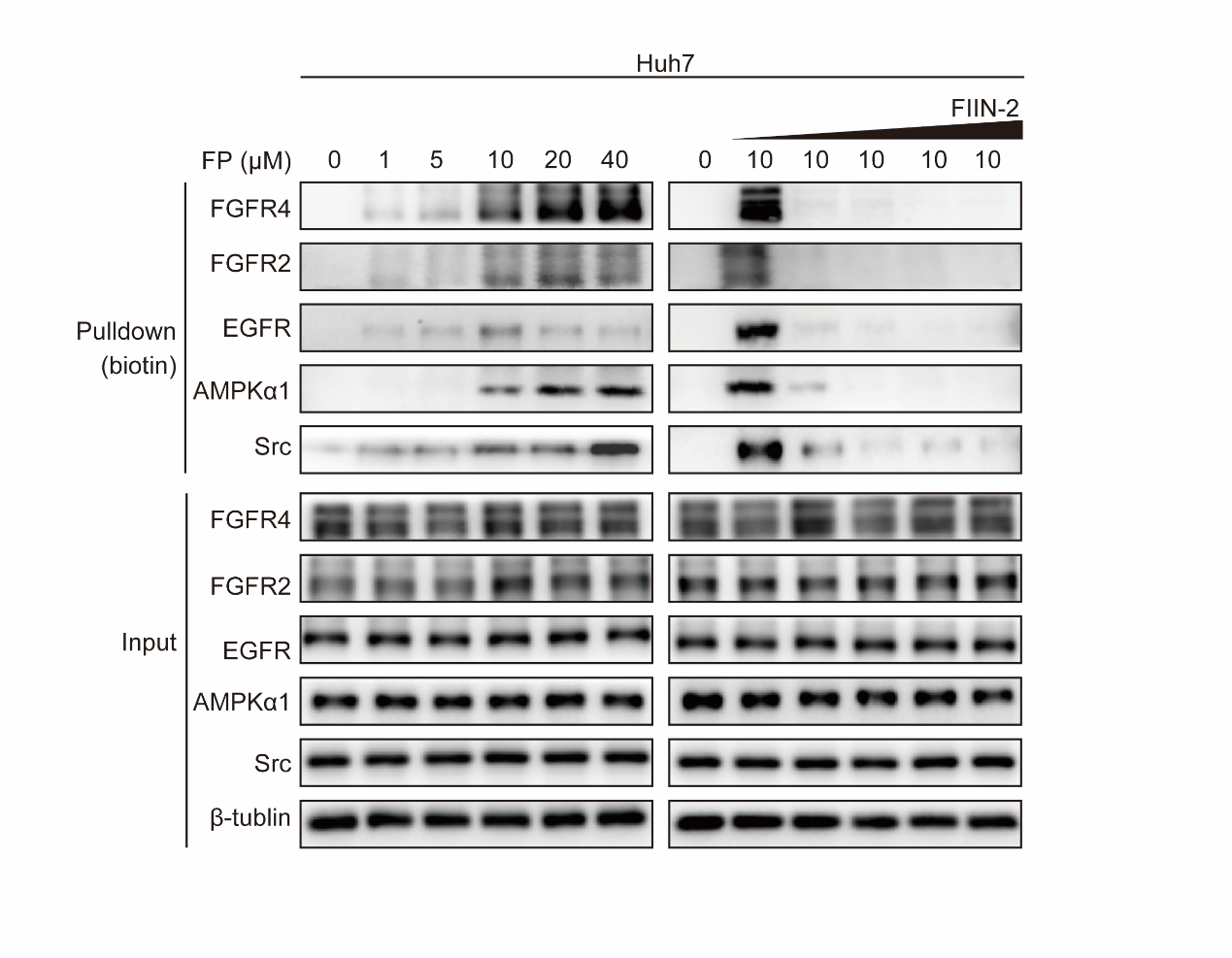
**

**Figure S3.** Pull-down validation of FIIN-2-targeted proteins in Huh7 cells.

**
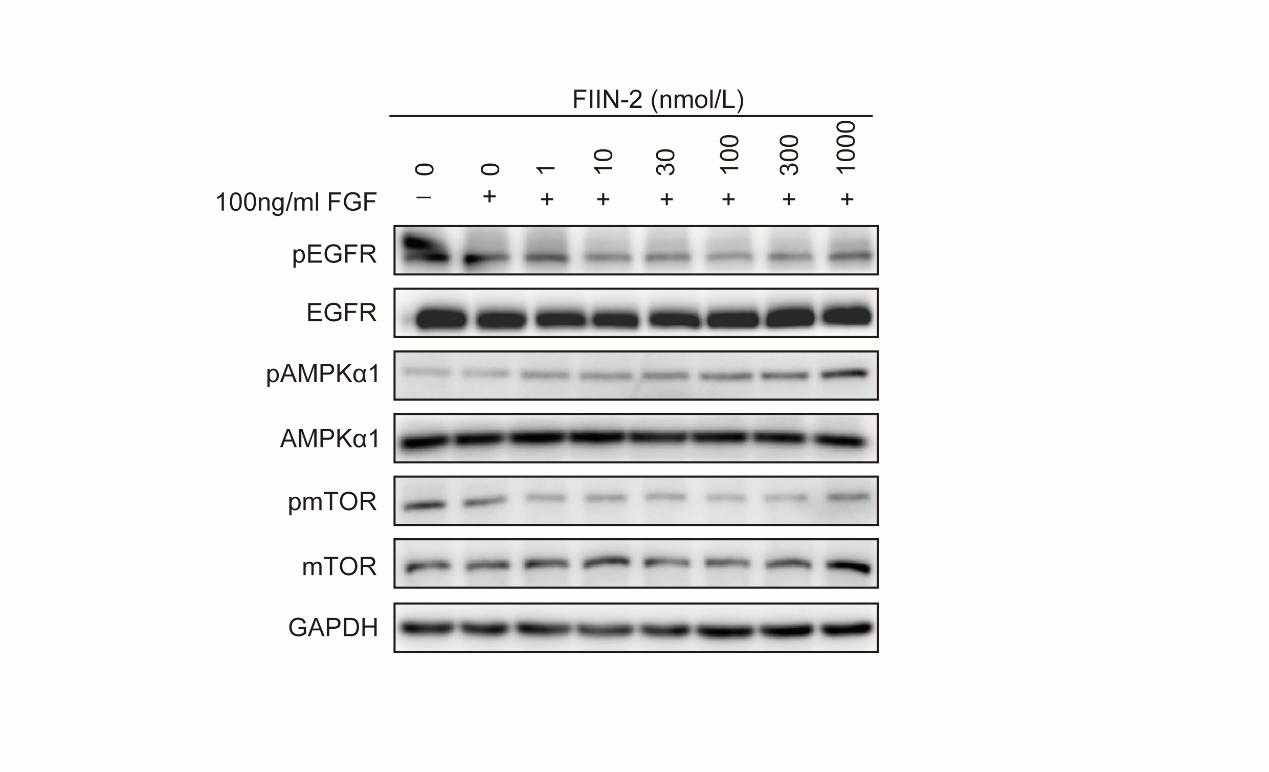
**

**Figure S4.** Validation of the differential phosphoproteins identified by phosphoproteomics via western blotting in Huh7 cells.

**
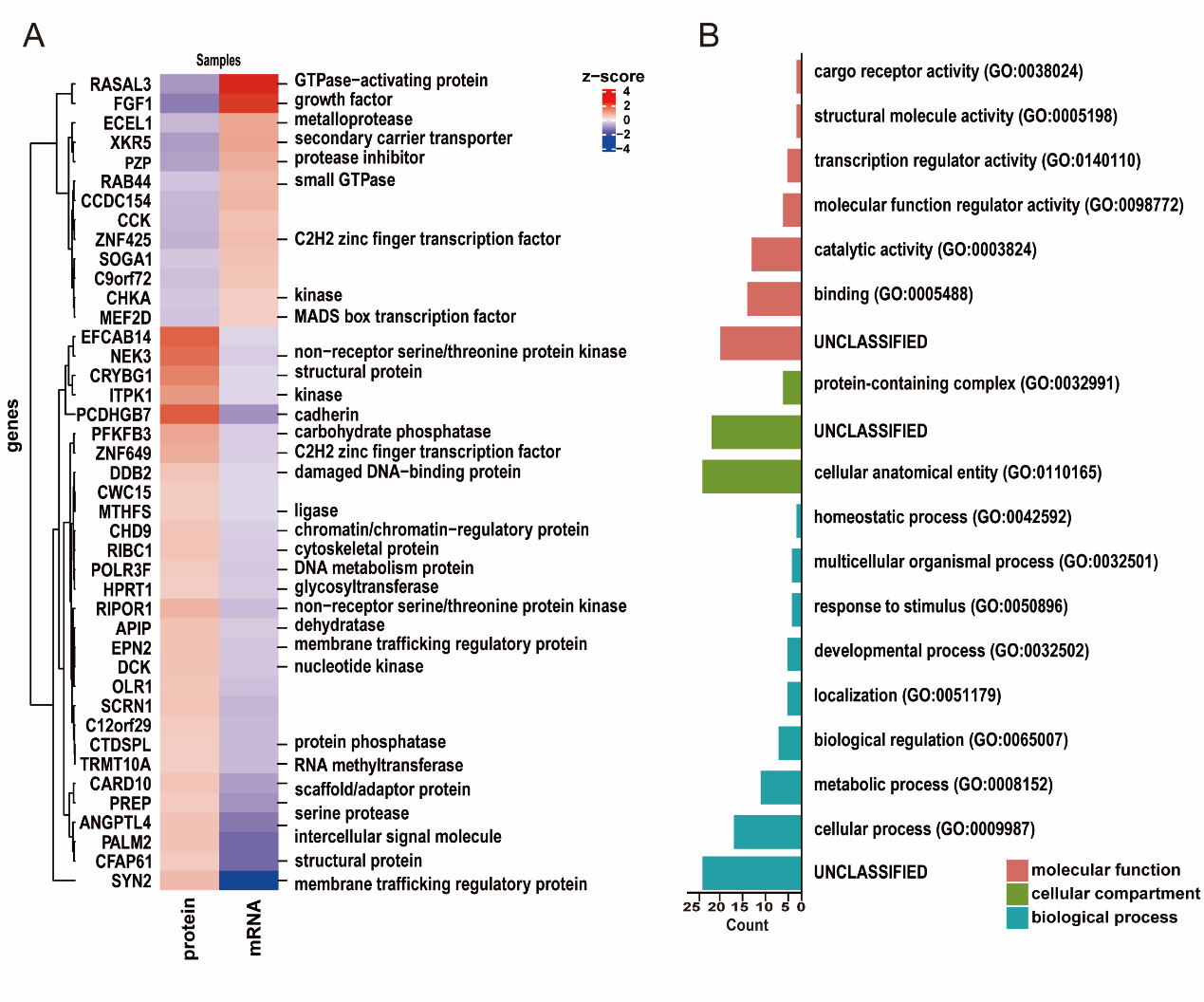
**

**Figure S5. Bioinformatic analysis of genes whose mRNA and protein expression levels were inversely correlated.** (A) Heatmap of these genes. (B) Gene ontology annotation of these genes.

**
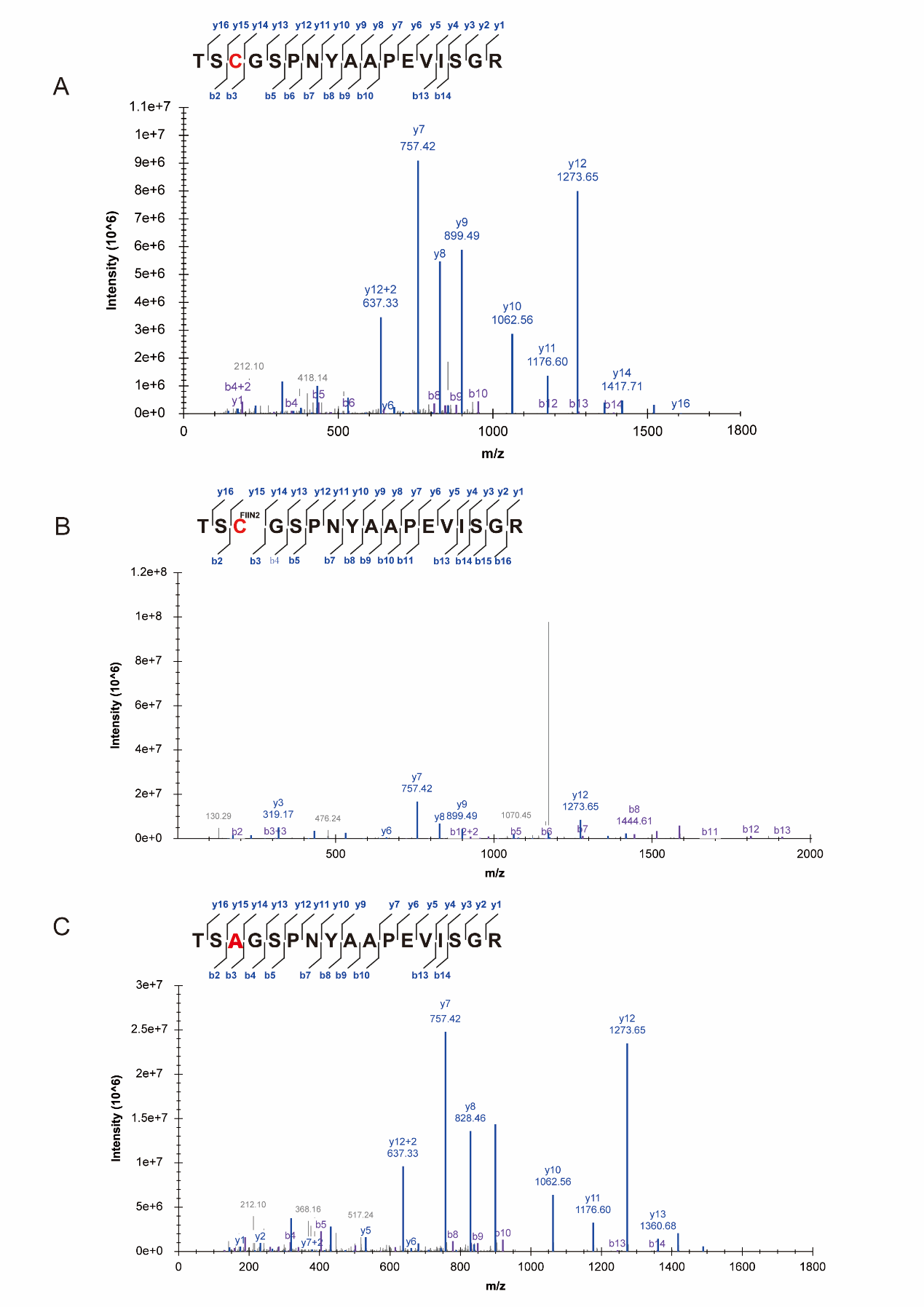
**

**Figure S6. Detection of in vitro covalent peptide–drug adducts via LC‒MS/MS.** (A) Fragment spectra corresponding to the peptide TSCGSPNYAAPEVISGR containing a C185 site from AMPKɑ1 after DMSO treatment. (B) Fragment spectra corresponding to the peptide TSC(FIIN2)GSPNYAAPEVISGR from AMPKɑ1 after FIIN2 treatment. (C) Fragment spectra corresponding to the peptide TSAGSPNYAAPEVISGR from C185A mutant AMPKɑ1 after FIIN2 treatment.

**
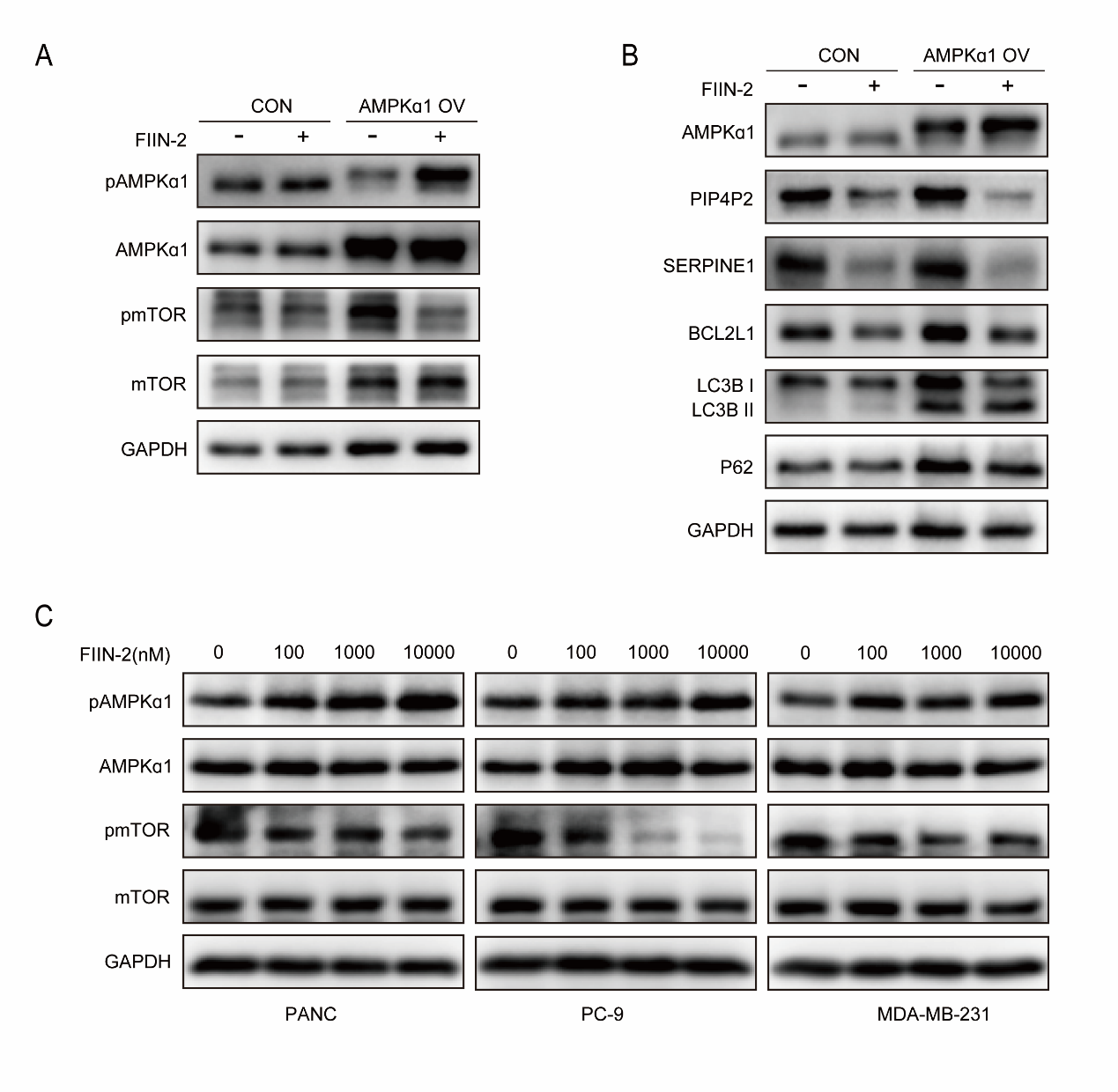
**

**Figure S7. The role of AMPKɑ1 in the function of FIIN-2.** (A) Overexpression of AMPKɑ1 enhanced the impact of FIIN-2 on its downstream signaling pathway. (B) Overexpression of AMPKɑ1 promoted the regulatory effect of FIIN-2 on its downstream proteins (PIP4P2, SERPINE1 and BCL2L1) and its effect on autophagy (P62 and LC3B). (C) FIIN2 can activate the AMPK signaling pathway by increasing the phosphorylation of AMPK and inhibiting the phosphorylation of mTOR in multiple types of human cancer cells.

**
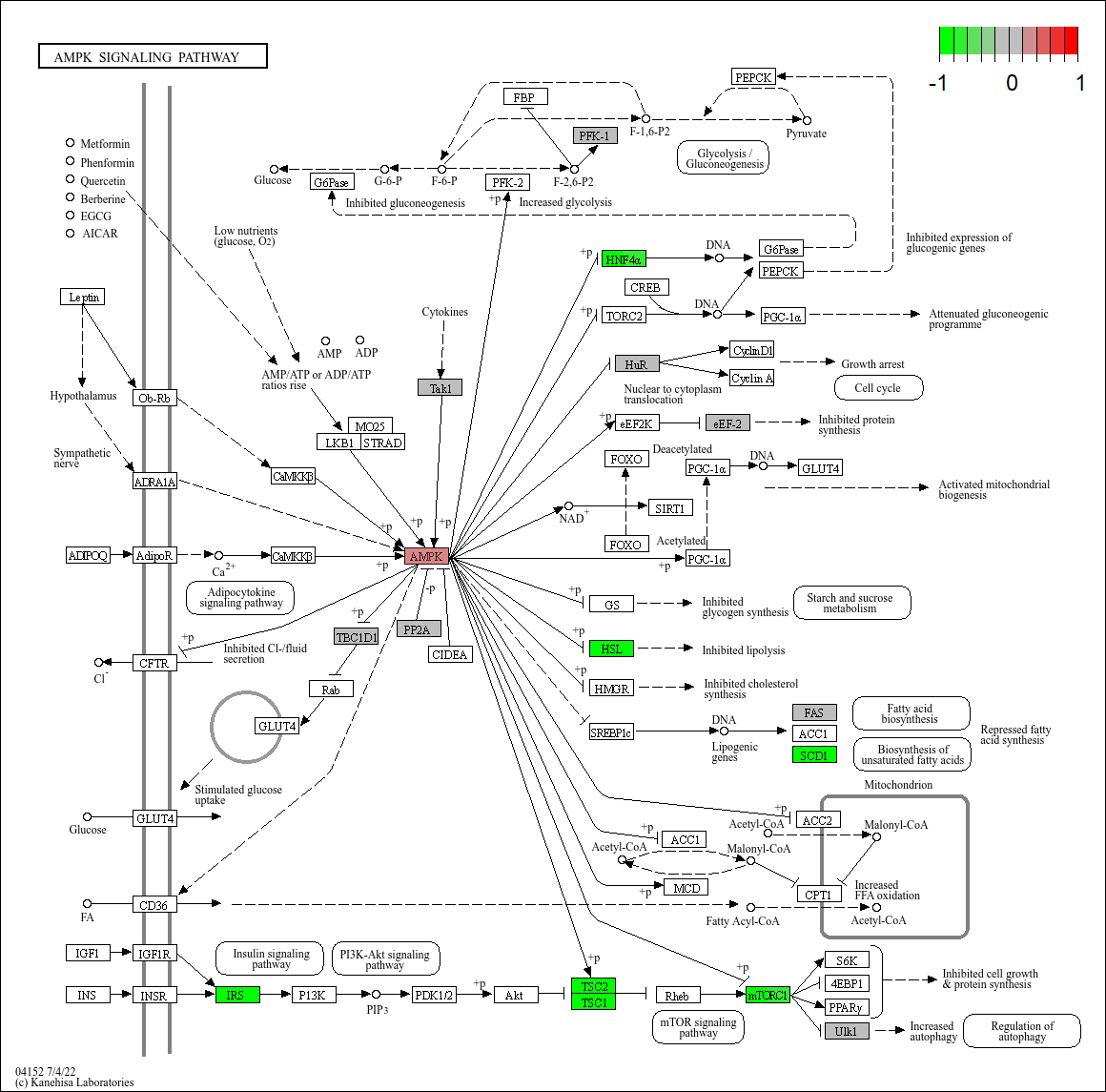
**

**Figure S8.** Changes in the phosphorylation of proteins in the mTOR signaling pathway induced by FIIN-2. The proteins in the red box indicate that the phosphorylation level was elevated, and the proteins in the green box indicate that the phosphorylation level was decreased.
