# Supplementary material for: Integrative Multi‐Omics Approaches Reveal Selectivity Profiles and Molecular Mechanisms of FIIN‐2, a Covalent FGFR Inhibitor
Source: Adv Sci (Weinh). 2025 Feb 20;12(14):2412578. doi: 10.1002/advs.202412578 (PMC11984845; doi:10.1002/advs.202412578)
Supplement: Supplementary file 8 — Supplemental Table S7 [file ADVS-12-2412578-s006.docx]

**Supplementary Table 7 List of the primers sequence**

| Gene name | Forward primer (5'-3') | Reverse primer (5'-3') |
| --- | --- | --- |
| CTGF | GTTTGGCCCAGACCCAACTA | GGCTCTGCTTCTCTAGCCTG |
| ICAM1 | ACGGAGCTCCCAGTCCTAAT | CTCCTTCTGGGGAAAGGCAG |
| EPHA2 | GATCGGACCGAGAGCGAGA | CAGTACCACTTCCTTGCCCT |
| CHPF | GCAACGACATCGTCAGTGCG | TATAGTGCACCCCCTCGTGGT |
| PIP4P2 | CTCTGGCAAAATGCCCACAC | CTTGCAAAATCTGGGGTGCC |
| SERPINE1 | CAGACCAAGAGCCTCTCCAC | CATGCGGGCTGAGACTATGA |
| BCL2L1 | AGGCGGATTTGAATCTCTTTCTCT | GGGCTCAACCAGTCCATTGT |
| β-actin | TGACGTGGACATCCGCAAAG | CTGGAAGGTGGACAGCGAGG |
